# Integrating brain organoids, meningeal immunity, and glymphatic dynamics: toward modeling neuroimmune clearance and crosstalk in disease

**DOI:** 10.3389/fimmu.2026.1931370

**Published:** 2026-08-24

**Authors:** Xiaolei Wu, Bin Du, Qian Chen, Ruyi Hang, Ce Cui, Mengxia Li

**Affiliations:** 1 Department of Cancer Center, Army Medical Center of PLA (Daping Hospital), Army Medical University, Chongqing, China; 2 Department of Neurology and State Key Laboratory of Biotherapy, National Clinical Research Center for Geriatrics, West China Hospital, Sichuan University, Chengdu, China

**Keywords:** brain organoids, glymphatic system, glymphatic-like transport, meningeal lymphatics, neuroimmune disease model, organ-on-a-chip

## Abstract

The central nervous system has long been considered an immune-privileged organ, but discoveries about meningeal lymphatics, meningeal immune cells, cerebrospinal fluid (CSF)-interstitial fluid (ISF) exchange, and aquaporin-4 (AQP4)-associated glymphatic-like transport are reshaping our understanding of waste clearance and immune surveillance in the brain. However, there are obvious breaks in the current model system. Animal models possess complete circulatory and meningeal structures, but are limited by species differences, imaging depth, operability, and high throughput. Traditional two-dimensional (2D) culture and transwell models lack 3D brain parenchymal structure, low-speed fluid dynamics, and the meningeal immune microenvironment. This paper proposes the concept of “brain parenchyma-lymphoid-meningeal immune assembly chip”, which integrates brain organoids containing neurons, astrocytes and microglia, CSF-like fluid derived from choroid plexus organoids, microfluidic lymphatic channels, and meningeal immune modules containing meningeal lymphatic endothelium, meningeal fibroblasts, macrophages, and dendritic cells. Currently, the various component technologies required for this platform are still at different stages of experimental maturity and have not yet been integrated into a single system. This review discusses existing issues such as the cellular composition of the brain parenchyma module, the engineering simulation of glymphatic dynamics, the reconstruction of the meningeal lymphatic and immune interface, disease applications, and validation standards. It also outlines a phased development roadmap to build a platform capable of tracking the continuous process of “pathological product generation-fluid clearance-meningeal immune sensing-inflammatory feedback” under simulated human brain conditions, providing new tools for mechanism analysis and drug screening in neurodegenerative diseases, brain injury, and neuro-immunological diseases.

## 1 Introduction

**Figure 1:**
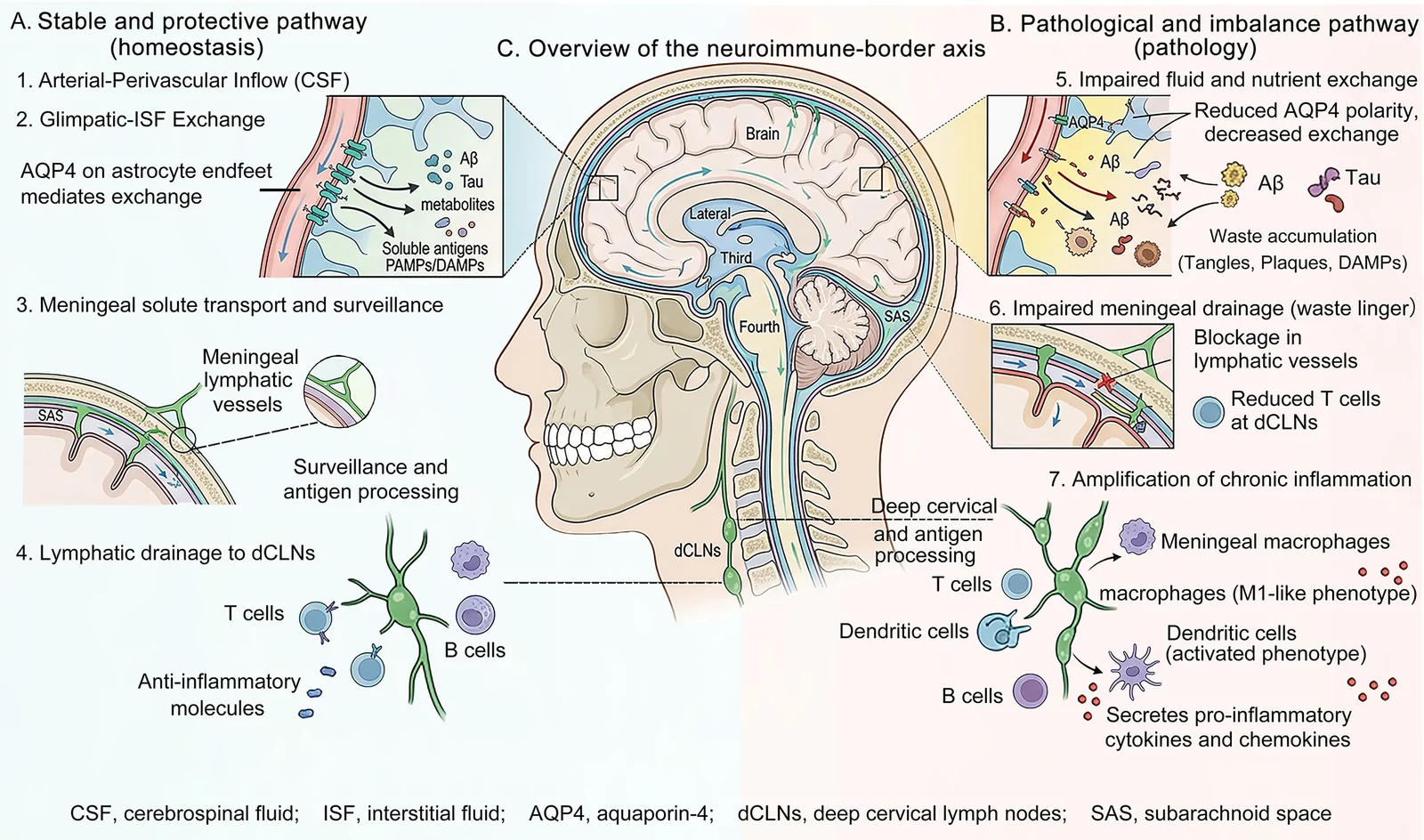
Brain parenchymal clearance and meningeal immune surveillance constitute a continuous neuroimmune boundary axis. The left side **(A)** shows the protective clearance pathway under homeostatic conditions: cerebrospinal fluid (CSF) enters the brain tissue along the periarterial space, where it exchanges with the interstitial fluid with the astrocyte terminale and aquaporin-4(AQP4), thereby propelling Aβ, Tau, metabolic waste, damage-associated molecular patterns (DAMPs), and soluble neuroantigens toward the meningeal boundary. Subsequently, meningeal lymphatic vessels drain these brain-derived molecules to the deep cervical lymph nodes, working with meningeal immune surveillance to maintain brain homeostasis. The right side **(B)** shows the imbalance under pathological conditions: decreased AQP4 polarization, weakened lymphoid fluid exchange, or impaired meningeal drainage can lead to the retention of pathological products in the brain parenchyma, prolonged meningeal exposure time, and further induce sustained activation of meningeal macrophages and dendritic cells, amplifying the local inflammatory response. The sagittal outline of the skull in the middle **(C)** emphasizes that lymphoid transport and meningeal immunity are not isolated systems, but should be understood as a functionally coupled brain boundary neuroimmune clearance axis.

For a long time, the central nervous system (CNS) has been considered an immune privileged organ relatively isolated from the peripheral immune system, with its homeostasis mainly relying on the protection of the blood-brain barrier and local regulation by neurons and glial cells within the brain tissue. However, research over the past decade has gradually changed this traditional understanding. Increasing evidence suggests that the CNS is not a completely closed system, but rather continuously exchanges information and transports substances with the peripheral immune system through the meninges, choroid plexus, perivascular spaces, cerebrospinal fluid (CSF) pathways, and skull-bone-associated niches (1–3). In particular, the rediscovery of dural lymphatic vessels and the introduction of the concept of glymphatic clearance and meningeal lymphatic drainage have led to a renewed understanding of the mechanisms for clearing brain waste, soluble antigens, and inflammatory mediators (4, 5). In the new framework, the lymphoid system and meningeal immunity are not viewed as two separate research areas, but rather as upstream and downstream links in the same continuous neuroimmune clearance axis. CSF enters brain tissue via the periarterial pathway, where it exchanges with interstitial fluid (ISF) with the participation of aquaporin-4 (AQP4) enriched astrocyte terminale, transporting metabolic waste, protein aggregates, inflammatory signals, and neuroantigens towards the perivenous or meningeal border (4, 6–8). After entering the meninges, these molecules are further exposed to the boundary immune niche composed of lympho-endothelial cells, macrophages, dendritic cells, T cells, B cells, and fibroblasts, where they are taken up, processed, presented, or transported (5, 9, 10). Therefore, lymphoid fluid transport determines whether brain-derived pathological signals can reach the boundary, while meningeal immunity determines whether these signals are ultimately interpreted as homeostatic clearance, immune tolerance, or chronic inflammation and pathological amplification (**Figure 1**). This continuum is crucial in a variety of neurological diseases. In Alzheimer’s disease (AD), impaired clearance of β-amyloid (Aβ) and pathological Tau, AQP4 polarization disorder, and decreased meningeal lymphatic function collectively promote the accumulation of pathological proteins and the maintenance of chronic inflammation (7, 11). In traumatic brain injury (TBI), mechanical damage can induce the massive release of damage-associated molecular patterns (DAMPs) such as adenosine triphosphate (ATP), high mobility group box 1 (HMGB1), and S100 calcium-binding protein β (S100β), accompanied by changes in hydrodynamics and abnormal AQP4 localization, thereby enhancing the spread of inflammation at the brain boundary (12–14). In neuro-infections and autoimmune encephalitis, virus-associated molecules, inflammatory mediators, and neuronal autoantigens may also enter the meningeal immune zone through CSF-related pathways, participating in local myeloid cell activation and subsequent adaptive immune events (15, 16). Therefore, an increasing number of neurological diseases should not be understood merely as focal lesions within the brain parenchyma, but should be redefined as the result of a continuous imbalance between pathological signal generation, fluid transport, and boundary immune perception.

**Figure 2:**
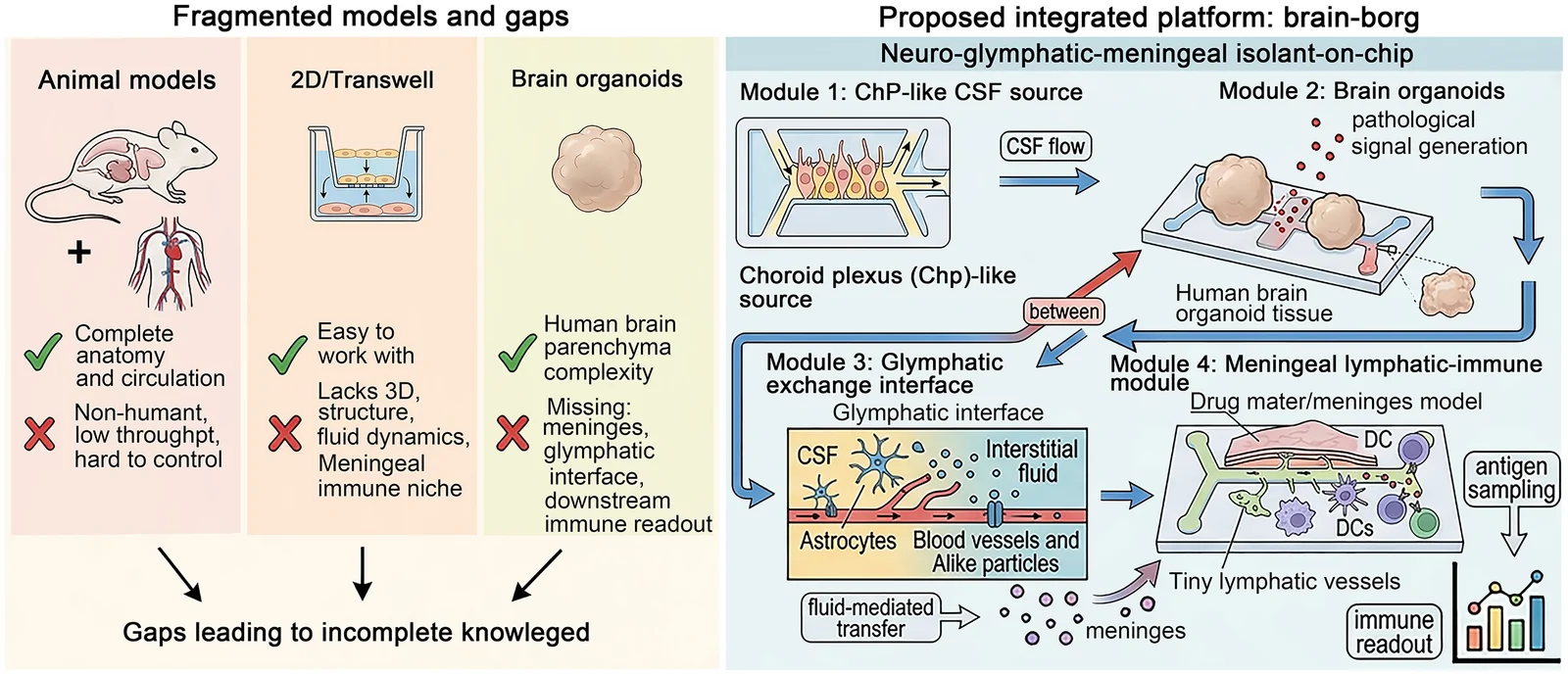
From fragmented models to brain parenchyma-lymphoid-meningeal immune assembly chip platform. This figure summarizes the main limitations of existing research models in analyzing the neuroimmune clearance process at the brain boundary. The left side shows the gaps in three commonly used models: animal models, while preserving complete anatomical structures and circulatory systems, suffer from species differences, limited controllability of genetic background, and insufficient high-throughput applications; two-dimensional culture and transwell systems are easy to manipulate but lack three-dimensional brain tissue construction, low-speed fluid transport, and a meningeal immune microenvironment; brain organoids, while capable of reproducing the diversity of human brain parenchymal cells and the genetic background of diseases, typically lack directed CSF-like input, meningeal lymphatic drainage, and downstream immune readout. The right side shows the humanized integration platform proposed in this paper, which consists of a choroidal CSF source module, a brain organoid module, a microfluidic exchange interface, and a meningeal lymphoid-immune niche module. This platform aims to integrate the generation of brain parenchymal pathological signals, fluid-mediated transport, and meningeal antigen sampling and immune response into a single dynamic system.

Although this theoretical picture is becoming increasingly clear, current experimental models still struggle to fully reconstruct this continuous axis within the same system. Animal models possess complete structures and circulation, but are limited by species differences; while two-dimensional (2D) culture and transwell systems are easy to manipulate, they lack three-dimensional (3D) brain tissue structures and realistic fluid environments (17). Human brain organoids can reproduce regional neural phenotypes and disease-related functions, and there have been reports that organoids contain microglia or blood vessels (18, 19), which gives them a unique advantage in human development and disease modeling. However, they typically lack directional CSF-like flow, meningeal boundaries, and downstream immune sampling systems, while existing on-chip organoid models mostly focus on a single interface (20–24). Choroid plexus organoids can form selective barriers and secrete CSF-like fluids, while on-chip glial vessel and lymphatic vessel models can generate directional ISF flow and quantify the transport of AQP4-related tracers (22, 25, 26). The lymphatic vessels and brain-meningeal co-culture system on three-dimensional chips further reproduced specific lymphatic or matrix functions (27). However, these findings are still scattered across different experimental systems, so we also summarize the current status and readiness of *in vitro* model components here (**Table 1**). Currently, there is no human *in vitro* model that can integrate the choroid plexus, neuronal-astrocytic-microglia parenchymal tissue, directed CSF-ISF exchange, and meningeal lymphatic-immune microenvironment into a well-balanced circuit that produces CSF. This raises the question of how to simulate the process by which pathological proteins, DAMPs, and inflammatory mediators generated in the brain parenchyma are transported to the meningeal boundary via the glymphatic pathway, and further transformed into a measurable meningeal immune response *in vitro*. Based on this question, we propose a conceptual framework of a “brain parenchyma-lymphoid-meningeal immune assembly chip”, advocating the integration of a choroid plexus-like CSF-derived module, a brain organoid module, a lymphoid-like microfluidic exchange interface, and a meningeal lymphoid-immune module into a continuous humanized platform (**Figure 2**). Compared with existing organoid or organ-on-chip systems that usually model only one interface, this platform is intended to capture the sequential process from parenchymal pathological product release to meningeal immune activation. Next, we will discuss the platform’s biological basis, engineering design logic, disease application scenarios, and future development direction in turn.

**Table 1:** The current status of the proposed platform components, the tested features, and the unresolved requirements.

Proposed component	Current models in *in vitro*	Experimentally demonstrated functions	Engineering implementation status	Missing functions and major modeling challenges
Choroid plexus/CSF source module	Human pluripotent stem cell derived choroid plexus organoids (22)	Formation of choroid plexus-like epithelium; selective barrier properties; prediction of small molecule CNS permeability; secretion of protein containing CSF-like fluid into self-contained lumens (22)	While existing biotechnologies can achieve this goal, significant fluid modification is still required. Existing choroid plexus organoids can provide barrier and secretory phenotypes; however, achieving controlled luminal pathways and continuous cerebrospinal fluid-like outflow requires *de novo* interface development (22)	Controlled access to the enclosed lumen; continuous collection of secreted fluid; quantitative measurement of secretion rate, pressure and composition; adult or disease-specific maturation; stable coupling to a downstream microfluidic circuit
Brain parenchymal source module	Regionally patterned cerebral organoids (21); organoids containing endogenous or introduced microglia (18, 52); organoids containing vascular-like structures (29)	Reproduction of cortical cell diversity (21); microglial differentiation, phagocytosis, inflammatory responses and microglia neural interactions (18, 52); vascular-like networks, tight junction associated phenotypes and improved organoid maturation (29)	This method can be implemented using existing organoid and co-culture techniques, but further optimization is still needed. Brain organoids, microglia integration, and vascular-like engineering protocols already exist, but adult-like maturation, standardized cell composition, controllable effluent acquisition, and reproducible AQP4 polarization are still insufficient (18, 21, 29, 52)	Simultaneous adult-like maturation of neurons, astrocytes and microglia; reproducible cell type ratios; controlled collection of pathological products; prevention of hypoxic cores; stable polarization of astrocytic AQP4 toward a perfusable fluid or vascular interface
CSF-ISF/glymphatic exchange module	Human gliovascular unit on chip and glymphatics on chip models containing astrocytes, endothelial channels and 3D extracellular matrix (25, 26)	Formation of astrocytic endfoot vascular interfaces; AQP4 polarization; directional interstitial fluid transport; quantitative tracer and Aβ drainage; impairment of transport after inflammatory stimulation, AQP4 inhibition or Aβ exposure (25, 26)	Microfluidic fabrication and directional-flow technologies are currently available. Adaptation is required to accommodate intact organoids, physiological low-flow conditions, pulsatility and quantitative mass balance; the organoid-fluid interface itself requires substantial new development (25, 26, 72, 73, 77, 78)	Authentic periarterial to perivenous organization; physiological pulsatility and very low velocity flow; incorporation of neurons and microglia; coupling to a CSF-producing source and a downstream meningeal lymphatic outlet; quantitative distinction among diffusion, convection, cellular uptake, degradation and matrix adsorption
Meningeal lymphatic endothelial module	Three-dimensional human lymphatic vessel on chip models (73); simplified meningeal cell lymphatic endothelial co-culture models (92)	Lymphatic endothelial tubularization; responses to interstitial flow and VEGF-A/VEGF-C; lymphatic sprouting and discontinuous junction formation (73); formation of lymphatic endothelial and meningeal cell layers and measurement of dextran permeability in a meningeal specific co-culture (92)	While general-purpose on-chip lymphatic technology has been developed, its specific application to the meninges is still in its early stages. Constructing a perfusionable human meningeal lymphatic network with cerebrospinal fluid absorption and immune cell transport capabilities requires extensive modifications or *de novo* biological reconstruction (73, 92)	Stable, perfusable human meningeal lymphatic networks under CSF-like flow; preservation of meningeal lymphatic endothelial identity; directional uptake of macromolecules; integration with fibroblasts and immune cells; measurement of antigen and immune-cell trafficking
Meningeal fibroblast myeloid immune niche	Human cortical organoids encapsulated with human meningeal cells (27); human neural organoids fused with mouse fetal leptomeninges containing fibroblasts and macrophages (97)	Meningeal coverage of cortical organoids; improved cortical organization and astrocyte formation (27); maintenance of layer associated meningeal fibroblasts and resident macrophages and formation of a stable neural meningeal interface for up to 60 days (97)	Individual cell sources and brain meningeal co-culture methods are available, but the proposed multicellular functional module has not been established. Spatial organization of lymphatic endothelium, fibroblasts, macrophages and dendritic cells, together with measurable antigen processing and immune activation, requires near *de novo* development (27, 97)	Fully human and spatially organized meningeal tissue; simultaneous incorporation of meningeal lymphatic endothelium, fibroblasts, macrophages and dendritic cells; functional antigen uptake, processing and presentation; cytokine and chemokine responses linked quantitatively to defined upstream brain derived signals
Fully integrated brain parenchyma lymphoid meningeal immune platform	Relevant functions have been demonstrated separately in choroid plexus organoids, brain organoids, glymphatics on chip, lymphatic vessel on chip and brain-meningeal co-culture systems (18, 22, 25–27, 29, 52, 73, 92, 97), but not in one continuous human *in vitro* circuit	Individual upstream pathological-signal generation, CSF-like secretion, AQP4 associated transport, lymphatic endothelial transport and selected meningeal stromal interactions have been demonstrated in separate systems	Simplified modular prototypes can be achieved using existing microphysiological systems technologies. However, complete products require development from scratch of standardized modular connectors, cross-module culture conditions, synchronized operating windows, quality balance analysis, and system level validation	Cross module medium and ECM; synchronized maturation; physiologically scaled flow and compartment volumes; quantitative mass balance; long-term functional stability; separation of true clearance from adsorption or degradation; validation against human CSF, tissue and imaging data

*De novo* development refers to platform-specific biological and fluid integration, rather than inventing a new fundamental microfabrication method. These assessments represent a comprehensive analysis of the cited literature by the authors, rather than a classification assigned by the original investigators.

## 2 Brain organoids as sources of pathological and immune signals in brain parenchyma

**Figure 3:**
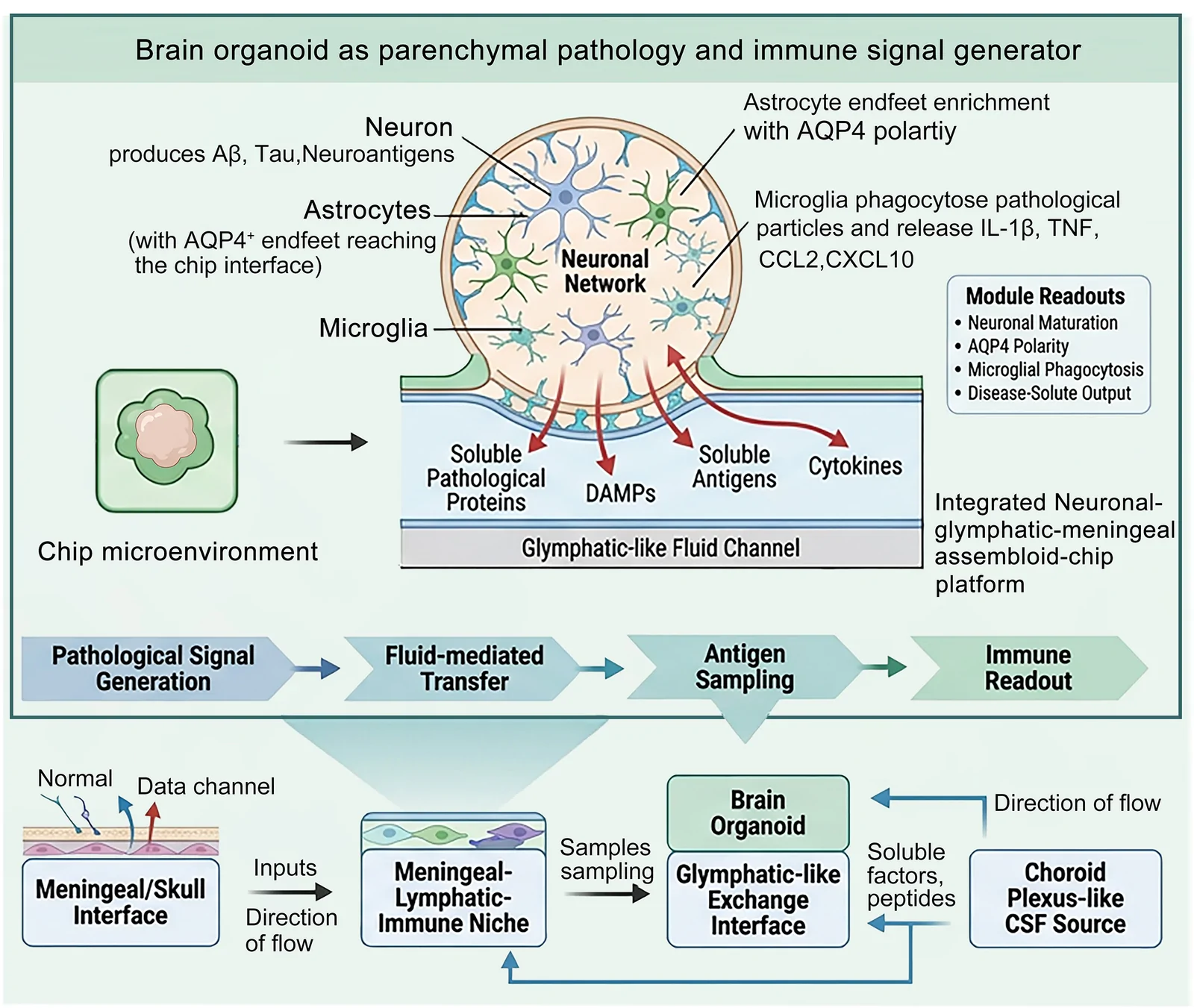
Brain organoids as upstream generators of brain parenchymal pathology and immune signals. This figure illustrates the brain parenchyma module within the integrated platform. The central brain organoid comprises an interconnected network of neurons, maturing astrocytes, and functional microglia. Neurons produce disease-associated pathological products such as Aβ, Tau, and soluble neuroantigens; astrocytes extend their terminals to the perivascular space-like interface and form AQP4-enriched structures, providing the tissue basis for subsequent lymphoid CSF-interstitial fluid (ISF) exchange; microglia transform local pathological changes into propagating immune perturbations by phagocytosing pathological particles, activating inflammasomes, and releasing factors such as interleukin (IL)-1β, tumor necrosis factor (TNF), chemokine ligand 2 (CCL2), and C-X-C motif chemokine ligand 10 (CXCL10). Red arrows represent pathological proteins, DAMPs, and soluble antigens exported from the brain parenchyma module; these molecules will enter the downstream fluid interface and determine the antigen exposure pattern on the meningeal side. The figure also indicates key functional readouts of this module, including neural network maturity, AQP4 polarization, microglia phagocytic activity, and the ability to export pathological molecules.

In the platform proposed in this paper, the role of brain organoids is not merely to provide a 3D tissue model that approximates the human brain development process, but more importantly, it must serve as an upstream source of brain parenchymal pathological products and innate immune signals (18, 28, 29) (**Figure 3**). At the component level, this part of the platform has the most solid experimental foundation. Human brain organoids containing neurons and astrocytes have been routinely constructed, and endogenously or exogenously introduced microglia can exhibit branching, phagocytosis, inflammatory responses, and disease-related phenotypes (30–32). Furthermore, there have been reports of vascular-like structures and neurovascular aggregates (33, 34). However, no organoid model currently provides simultaneously adult-like neuronal and glial cell maturation, stable microglial homeostasis, polarized astrocytes AQP4 at the perfusion interface, controlled collection and release of pathological products, and low batch-to-batch variability. Therefore, simply including a specific cell type is insufficient to constitute an adequate condition for constructing a clearance platform. A suitable brain organoid for inclusion in a neuroimmune clearance platform should not only resemble the brain, but should be able to stably produce disease-related Aβ, Tau, DAMPs, soluble neuroantigens, and glial-derived inflammatory signals, and enable these signals to be transported by subsequent fluid interfaces and perceived by the meningeal immune system. From a functional perspective, an ideal brain parenchyma module should include at least three key cell types. First, a relatively mature population of neurons, which determines the continuous production of pathological proteins and synaptic-related molecules (35). Second, sufficiently mature astrocytes with AQP4 expression potential, which not only support brain tissue homeostasis but also form the important structural basis for subsequent glymphatic-like CSF-ISF exchange (6, 25, 36, 37). Third, functional microglia, which transform pathological changes within the brain parenchyma into transmissible immune perturbations through phagocytosis, complement-related pruning, inflammasome activation, and chemokine release (18, 38, 39). Therefore, the role of brain organoids in this integrative system is essentially that of a source of pathological signals rather than a simple developmental model. The requirements for regionalization and maturity of brain organoids vary depending on the disease context. Cortical or hippocampal organoids are more suitable for AD to study Aβ, Tau, synaptic loss, and microglia phagocytosis (21, 40); cortical organoids, assembloids containing long-range axonal tracts, or brain tissue-like models that can be subjected to mechanical stress are more suitable for TBI (41); and choroid plexus-like components or barrier-related cells are more suitable for infection and autoimmune models to observe pathogen-related molecules and antigen expulsion processes (23, 42). At the same time, the maturity criteria for organoids should not rely solely on morphological indicators, but should be comprehensively evaluated in combination with neural network activity, synaptic protein expression, glial cell maturation status, and pathological output profiles. Maturity may be assessed by synaptic markers such as synapsin 1 (SYN1) and postsynaptic density protein 95 (PSD95), astrocytic markers such as glial fibrillary acidic protein (GFAP) and S100β, microglial markers such as transmembrane protein 119 (TMEM119), purinergic receptor P2Y12 (P2RY12) and ionized calcium-binding adapter molecule 1 (IBA1), and functional readouts such as calcium activity or multielectrode array recordings (18, 43, 44). For integration into a clearance-immune microphysiological system, brain organoid quality should be defined prospectively. Minimum reporting requirements should include organoid diameter and tissue thickness, spatial viability and hypoxia-inducible factor-1 (HIF-1α) distribution, neuronal and astrocyte composition, microglia proportion, spontaneous and evoked electrophysiological activity, pathological product yield (normalized to tissue quality), and batch-to-batch variability (44–47). Since the diffusion of oxygen and nutrients is restricted in intact organoids (48–51), constructs with expanding hypoxic or necrotic cores should be sliced, placed near the perfusion interface, or supplied with blood through vascular-like channels, rather than being judged for their suitability solely based on external morphology.

In this platform, the integration of microglia is particularly crucial. They are not only resident immune cells within the parenchyma but also determine whether brain organoids truly possess neuroimmune properties (18, 52, 53). Endogenous microglia generation strategies are beneficial for developmental synchronization but exhibit significant batch-to-batch variability; exogenous implantation strategies are more suitable for patient-derived induced pluripotent stem cells (iPSCs) and gene-edited backgrounds (18, 54). Cell seeding should be determined based on the biological problem, regional characteristics, and stage of maturation, rather than using a universal ratio of neurons to glial cells. All studies should report the initial cell number, the proportion of mature cells, spatial distribution, and donor/batch variability, and these should be detected using methods such as quantitative imaging, flow cytometry, or single-cell analysis. As a practical benchmark, researchers have mixed 7,000 neural progenitor cells with 3,000 primitive macrophage progenitor cells and obtained about 8% microglia by day 50, close to the commonly cited proportion of microglia in the total cells of the central nervous system (5-10%) (55, 56). This value should be regarded as an initial benchmark for organoids containing microglia, rather than a mandatory proportion for each region or disease model. Regardless of the derivation strategy employed, the adequacy of microglia should be demonstrated through consistent evidence of identity, state, and function. TMEM119 supports microglia lineage/homeostatic identity (57), but its function remains undetermined; P2RY12 mediates nucleotide-guided protrusion extension and chemotaxis, and is often downregulated after activation (58); In addition to detecting the IBA1 biomarker, the identification of microglia may also require combining the activation status of the triggering receptor expressed on myeloid cells 2 (TREM2)-apolipoprotein E (APOE) pathway to determine the functional phenotype of microglia in neurodegenerative diseases (59). Functional validation should include live-cell monitoring or ATP/ADP chemotaxis (60), pulse-tracking uptake and degradation of labeled Aβ or Tau proteins, cytokine time progression, and pathway-selective perturbation (61–63). The NOD-like receptor pyrin domain-containing 3 (NLRP3) expression alone is insufficient to demonstrate inflammasome activation (64); apoptosis-related spot-like protein (ASC) speck formation, caspase-1 cleavage, and release of mature interleukin (IL)-1β are required to establish inflammasome activation (34, 65–68). Similarly, the significance of astrocytes in lymphoid interfaces lies not merely in their presence or absence, but in whether they form AQP4 polarized endplate structures. Many brain organoids express AQP4, but their distribution is often diffuse, lacking a spatial polarization relationship corresponding to the perivascular or fluid interface (25, 69). Therefore, future brain organoids used for neuroimmune clearance research should emphasize long-term maturation, co-culture with microvascular-like structures or interfaces, and directional polarization induction under microfluidic conditions (25, 26, 29, 37). AQP4 polarization is a key threshold for true coupling between brain parenchymal modules and lymphoid modules (70). However, it is important to distinguish AQP4 abundance from AQP4 polarization. The latter should be quantified as the ratio of AQP4 signal on the astrocyte membrane facing fluid or endothelial cells to the signal around the cell body/non-interface, and validated using orthogonal localization methods. Functional relevance requires demonstration that inhibition, knockdown, or controlled depolarization of AQP4 alters tracer transport without leading to nonspecific loss of tissue viability (71). Therefore, isotropic AQP4-positive organoids should not be considered validated glymphatic-like interface.

## 3 Engineered reconstruction of a lymphoid-mimetic interface for CSF-ISF exchange

**Figure 4:**
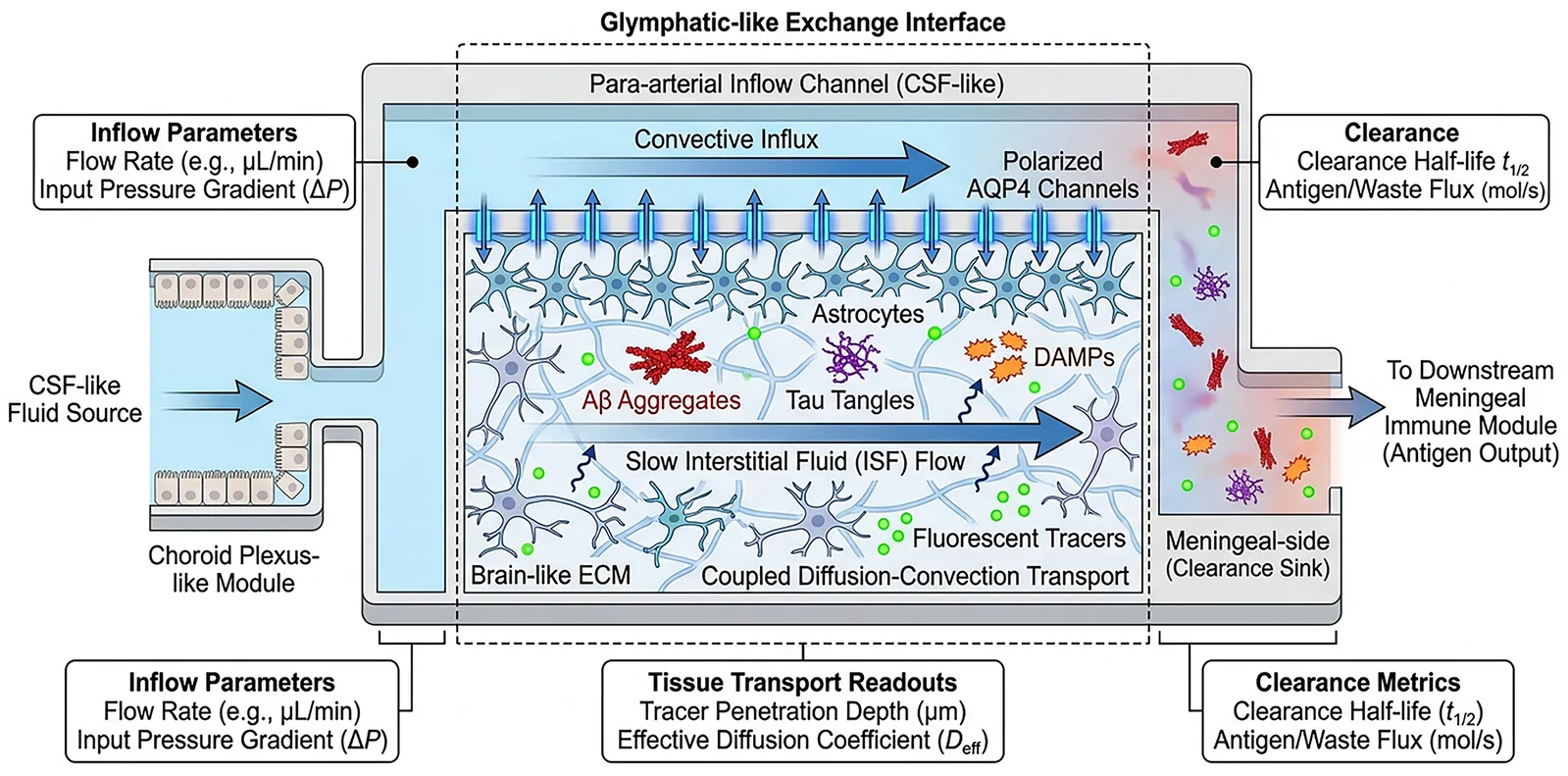
Engineering reconstruction of a glymphatic-like CSF-ISF exchange interface. This figure illustrates a lymphoid microfluidic module. The left choroid plexus-like module serves as the CSF-like fluid source, providing a continuous, low-velocity fluid inflow to the brain tissue side via perfusion channels. The central region contains brain-like extracellular matrix (ECM) and glial tissue areas, with AQP4-enriched astrocyte terminals visible at the tissue boundaries, supporting local exchange between CSF and interstitial fluid. Pathological molecules such as fluorescent tracers, β-amyloid (Aβ), Tau, or DAMPs can undergo diffusion-convection coupling transport at this interface, ultimately entering the right meningeal-facing effluent. This effluent serves as both a collection point for pathological molecules and an antigen input point for the downstream meningeal module. The figure also lists key engineering and functional readout parameters, including flow rate, pressure gradient, hydraulic resistance, tracer penetration depth, matrix permeability, tracer mass recovery rate, molecular size-dependent penetration, residence time, tissue deformation, and the clearance half-life of AQP4-perturbed pathological products, as well as the flux of antigen/pathological molecules in the effluent. This figure emphasizes that the lymphoid interface is not only a fluid connection structure within the entire platform but also a crucial control unit determining the dose and duration of meningeal immune exposure.

Even if brain organoids can produce pathological proteins, DAMPs, and inflammatory factors, their true boundary significance in disease remains difficult to elucidate if these signals cannot be delivered from the brain parenchyma to the meningeal boundary in a directional, slow, and quantifiable manner. Therefore, the construction of lymphoid interfaces is not about adding fluid to organoids, but about establishing a functionally defined pathological transport pathway that allows brain-derived pathological outputs to enter the downstream meningeal immune module in a measurable manner (22) (**Figure 4**). The platform should distinguish three fluid compartments: blood microcirculation, glymphatic-like CSF-ISF exchange, and meningeal lymphatic drainage. The first-generation design proposed in this paper simulates the latter two, but does not reproduce the real blood microcirculation. The glymphatic-like exchange module should include a pressure-controlled inflow channel, a brain tissue-like gel/tissue exchange area, and a separate sampling outflow channel (72, 73). The operating conditions of the device should be determined by measuring local flow rate, pressure drop, hydraulic resistance, matrix permeability, fluid viscosity, residence time distribution, tissue deformation, and tracer recovery, rather than solely by the pump setup. The amount of CSF produced in the adult brain is approximately 0.3-0.4 mL/min, which can provide a physiological background, but its size should not be reduced solely by volume to determine the ingress rate of the chip (74, 75). If brain microvascular channels are added, they should be validated as a separate vascular module; published iPSC-derived brain microvascular endothelial models have used shear stresses of 4 or 12 dyne/cm^2^ for 40 h (76), but these values should not be applied to parenchymal or lymphatic compartments. Furthermore, future versions should specify flow rate ranges, pressure gradients, matrix stiffness, pore size, and tracer molecular weights used to validate glymphatic-like transport. The upstream channel simulates CSF-like inflow around an artery, the adjacent area is filled with brain-like extracellular matrix (ECM) hydrogel containing brain organoids or glial cells, and the downstream channel collects solutes outflowing from the tissue area (77). The exchange interface can be achieved through a porous membrane or designed as an open boundary; porous membranes are easier to standardize, while open boundaries are closer to the real flexible tissue interface.

Material selection is crucial for result interpretation. Polydimethylsiloxane (PDMS) is easy to mold and has high transparency, but it has adsorption problems for certain hydrophobic small molecules and drugs (78). Therefore, for drug-screening applications, PDMS absorption of hydrophobic compounds should be controlled by using alternative materials or coating strategies; glass, cyclic olefin copolymer (COC), or modified polymers may be more advantageous in drug screening and quantitative exposure (79, 80). For the matrix, a complex system of collagen, hyaluronic acid, laminin, or brain-derived ECM is preferable to relying entirely on Matrigel, as matrix pore size, viscoelasticity, and adsorption characteristics directly affect the retention and output of pathological molecules in the tissue region (81). Human choroid plexus organoids derived from iPSC have reproduced several key functions of this module, including epithelial barrier selectivity, prediction of small molecule permeability, and secretion of protein-containing cerebrospinal fluid-like fluid into independent lumens (22, 82). Therefore, the biological feasibility of generating human cerebrospinal fluid-like sources has been sufficiently demonstrated (22). The remaining engineering challenges lie in the fact that the secreted fluid is confined within the lumen of the organoid and cannot be automatically obtained like a continuously perfused input (82). Integrating it into the proposed platform requires achieving controllable luminal access or cannulation, quantitatively measuring secretion rate and pressure, stabilizing cerebrospinal fluid composition, and demonstrating that the connection to downstream blood flow does not disrupt the properties of the choroid plexus epithelium (22, 23, 83). Transport validation should include tracers covering at least three molecular weight classes (for example, 3-10, 40, and 70 kDa), adsorption-corrected inlet and outlet mass balance, tissue penetration and retention, effluent appearance time, residence time distribution, and tissue clearance half-life (25, 26, 44). Actual flow rates and pressures should be validated on the device. A related 3D human lymphatic chip used an interstitial flow rate of 1 μm/s and a luminal shear stress of 3.5-4.5 dyne/cm² (73); these values can serve as initial benchmarks for engineering design, but are not established physiological values for human meningeal lymphatic vessels. We recommend using a total tracer recovery rate of ≥80% as the acceptable threshold for the detection method, explicitly defining it as an engineering quality control standard rather than a physiological constant. Causality should be tested by varying flow rate, hydraulic resistance, matrix permeability, or AQP4 polarization while monitoring tissue activity (25, 26, 71, 73, 84, 85). From an engineering perspective, the first-generation platform primarily utilizes existing microphysiological systems technologies. Soft lithography or micromilling can be used for rapid prototyping, while cyclic olefin copolymers or other thermoplastic materials are more suitable for later-stage device fabrication because these materials have higher requirements for small molecule adsorption, optical compatibility, and scalability. Standard perfusion pumps, pressure controllers, hydrostatic reservoirs, porous membranes, modular fluid connectors, and hydrogels containing collagen, hyaluronic acid, or laminin are sufficient to construct independent but fluidly connected compartments (72, 73, 77–81). For slowly maturing brain organoids and meningeal modules, replaceable inserts or cartridges should be used to culture biological samples separately, and connections should only be made after predefined quality control standards are met. Only when these engineering parameters and biological readouts establish a clear causal relationship can the lymphoid interface truly become a module with mechanistic research significance.

## 4 Reconstruction of the meningeal lymphatic immune niche *in vitro*

**Figure 5:**
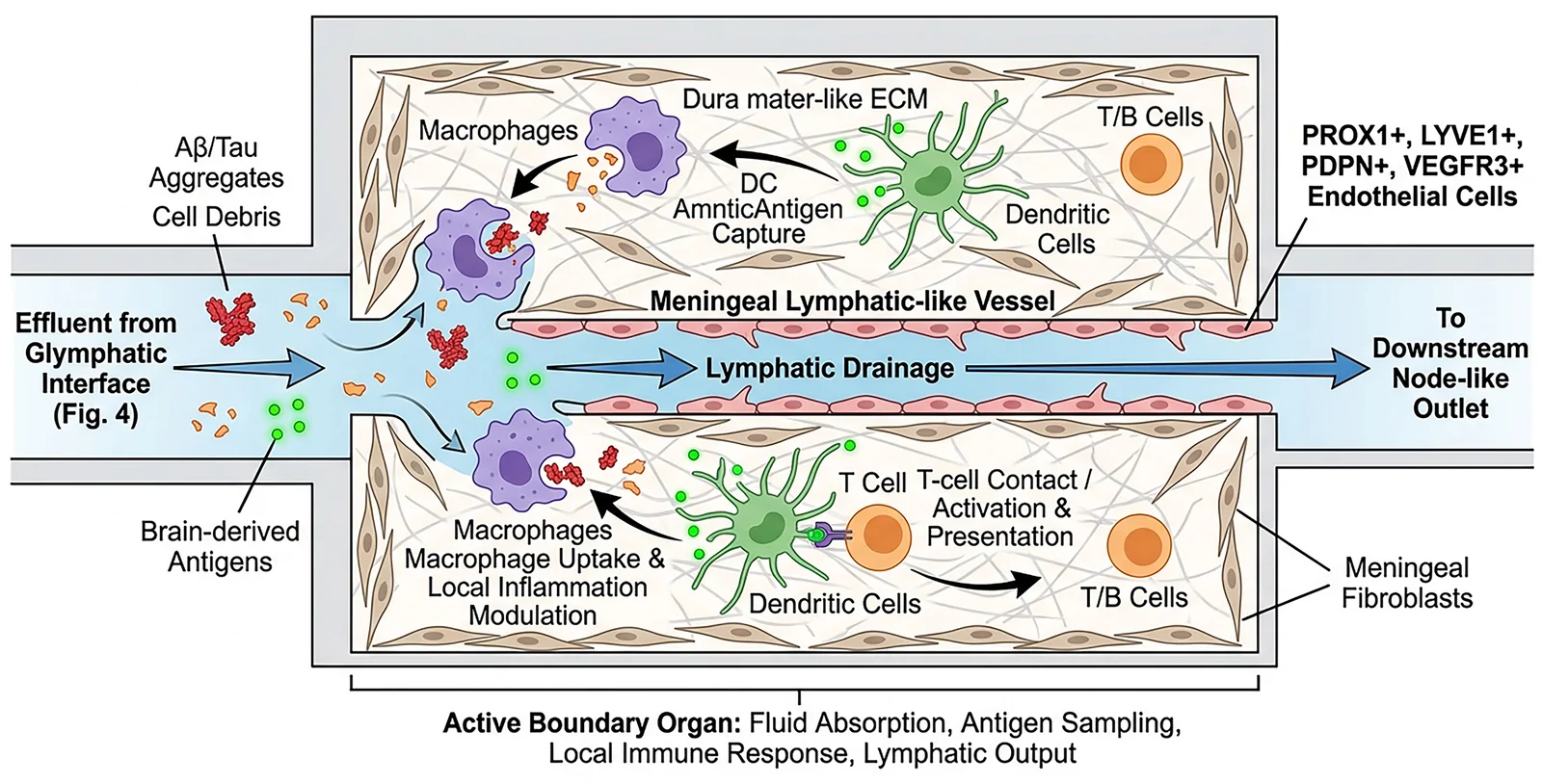
Reconstructing the meningeal lymphoid immune microenvironment *in vitro*. This figure illustrates the meningeal module within the integrated platform. The central area represents the dura mater-like stromal environment, containing meningeal lymphoid vessels formed by lymphoendothelial cells and a supporting matrix network composed of meningeal fibroblasts. The lymphoendothelial cells express typical markers such as prospero homeobox-1 (PROX1), lymphatic vessel endothelial hyaluronan receptor 1 (LYVE1), podoplanin (PDPN), and vascular endothelial growth factor receptor 3 (VEGFR3), reflecting their meningeal lymphatic phenotype. Importantly, PROX1, LYVE1, PDPN, and VEGFR3 support lymphatic endothelial cell (LEC) characteristics, while drainage requires lumen/connection analysis, permeability, uptake, downstream recovery, and VEGF-C/VEGFR3 perturbation. On the left, effluent from the lymphoid interface carries brain-derived antigens, pathological proteins, and cellular debris into the meningeal niche; macrophages can phagocytose these inputs and regulate local inflammation, while dendritic cells can take up antigens and present them to T cells, thereby promoting borderline immune activation. The lymphatic outflow direction on the right represents the drainage potential of the meningeal lymphatic vessels to more distal lymphatic structures. This figure emphasizes that the meningeal module should be viewed as an active border organ with functions encompassing fluid absorption, antigen sampling, local immune responses, and lymphatic outflow, rather than simply a fluid terminal.

In the integrated platform, the meningeal module is not simply a fluid receiver, but an active system with functions of boundary barrier, lymphatic absorption, antigen sampling, and immune regulation (**Figure 5**). The different layers of the meninges differ in cellular composition and function. The dura mater, rich in lymphatic vessels, blood vessels, fibroblast matrix, macrophages, dendritic cells, T cells, and B cells, is the most important functional region for brain boundary immune surveillance and lymphatic drainage (5, 10, 86–88). From a functional priority perspective, the most valuable area for *in vitro* reconstruction is not all meningeal layers, but rather representative dural-like lymphatic immune niches. Meningeal lymphatic vessels are one of the structural pillars of this module. *In vivo*, meningeal lymphatic vessels express typical markers such as prospero homeobox-1 (PROX1), lymphatic vessel endothelial hyaluronan receptor 1 (LYVE1) (89), podoplanin (PDPN) (90), and vascular endothelial growth factor receptor 3 (VEGFR3) (91), and participate in the drainage of fluids and macromolecules (3). Currently, the evidence for general lymphatic vessels is more robust than that for meningeal specific lymphatic vessels (92). 3D chip based lymphatic vessel models have demonstrated endothelial cell budding, discontinuous junction formation, permeability, and responsiveness to interstitial fluid flow and VEGF-C (93, 94). Recently, a simplified co-culture model of meningeal lymphatic vessels has been developed by mixing lymphatic vessel endothelial cells with meningeal cells and measuring dextran permeability (92). However, these systems have not yet been able to reproduce the perfusionable human meningeal lymphatic network exposed to cerebrospinal fluid-like fluid, fibroblasts, and antigen-presenting immune cells. Therefore, the main challenge lies not only in obtaining PROX1, LYVE1, PDPN, or VEGFR3-positive cells, but also in establishing the characteristics and targeted lymphatic function of the meninges in a relevant multicellular environment. In *in vitro* reconstruction, PROX1, PDPN, LYVE1, and VEGFR3 support the properties of lymphatic endothelial cells but do not by themselves demonstrate meningeal properties or drainage functions. Validation should also include patency of the lumen, connectivity, molecular size-dependent permeability, macromolecular uptake, downstream reabsorption, and consistent response to VEGF-C stimulation and VEGFR3 inhibition (1, 3, 73, 95, 96). Initial lymphoid discontinuous junctions should be distinguished from collecting vascular continuous junctions based on their intended absorption or transport function. Unless benchmarked against meningeal reference features, universal dermal lymphatic endothelial cells should be described as surrogate.

Currently, the maturity of matrix immune components is lower than that of brain organoid components. Human meningeal cells have been used to encapsulate cortical organoids, thereby improving cortical tissue structure and astrocyte formation, while leptomeningeal-neural organoid fusions can retain fibroblasts and resident macrophages (97, 98). These models provide proof of concept for a stable brain meningeal interface, but they primarily focus on developmental or nutrient signaling. Furthermore, these models have not yet demonstrated lymphoid uptake of brain-derived antigens, dendritic cell processing and presentation, or quantitatively related downstream immune responses. Therefore, the addition of these cells can only be considered functionally successful if macrophages or dendritic cells, upon exposure to specific upstream antigens, exhibit measurable uptake, processing, cytokine release, and, under appropriate conditions, cell migration or lymphocyte activation. Meningeal macrophages and dendritic cells determine whether pathological signals are truly interpreted as immune events (99). The meningeal immune module should first demonstrate a stable low-inflammatory baseline, including low spontaneous release of IL-1β, IL-6, TNF, CCL2, and CXCL10, as well as retained responsiveness to specific positive controls (100). Absolute cytokine values are device-dependent and should therefore be reported per live cell or tissue mass, along with the fold change from matched baseline and sampling dwell time. Macrophage validation should use pulse tracking assays and lysosomal readings to differentiate between uptake and degradation. For dendritic cells (101), major histocompatibility complex II (MHC-II) and CD80/CD86 indicate potential antigen-presenting capacity, while C-C chemokine receptor type 7 (CCR7) indicates migration capacity (5, 9, 102); functional validation requires antigen processing, CCL19/CCL21-mediated migration, and MHC-restricted activation of autologous T cells using irrelevant antigens, dendritic cells-free cells, and blocking controls (103, 104). Therefore, in the meningeal module, myeloid cells are not simply an add-on component, but rather a core node determining whether boundary exposure translates into immune readout. Meningeal fibroblasts and the ECM constitute the implicit framework of the meningeal niche (105, 106). Meningeal fibroblasts maintain the local mechanical environment, immune cell retention, and lymphoid structure stability by secreting components such as collagen, fibronectin, laminin, and hyaluronic acid (10, 107, 108). Without the support of these matrix components, meningeal modules are often just a cellular suspension and can hardly be considered true boundary tissue. Therefore, the construction of meningeal modules should emphasize the synergistic organization of lymphoendothelial cells, myeloid immune cells, and fibroblastic matrix, rather than a simple mixture of cells.

## 5 Disease modeling and translational applications

**Figure 6:**
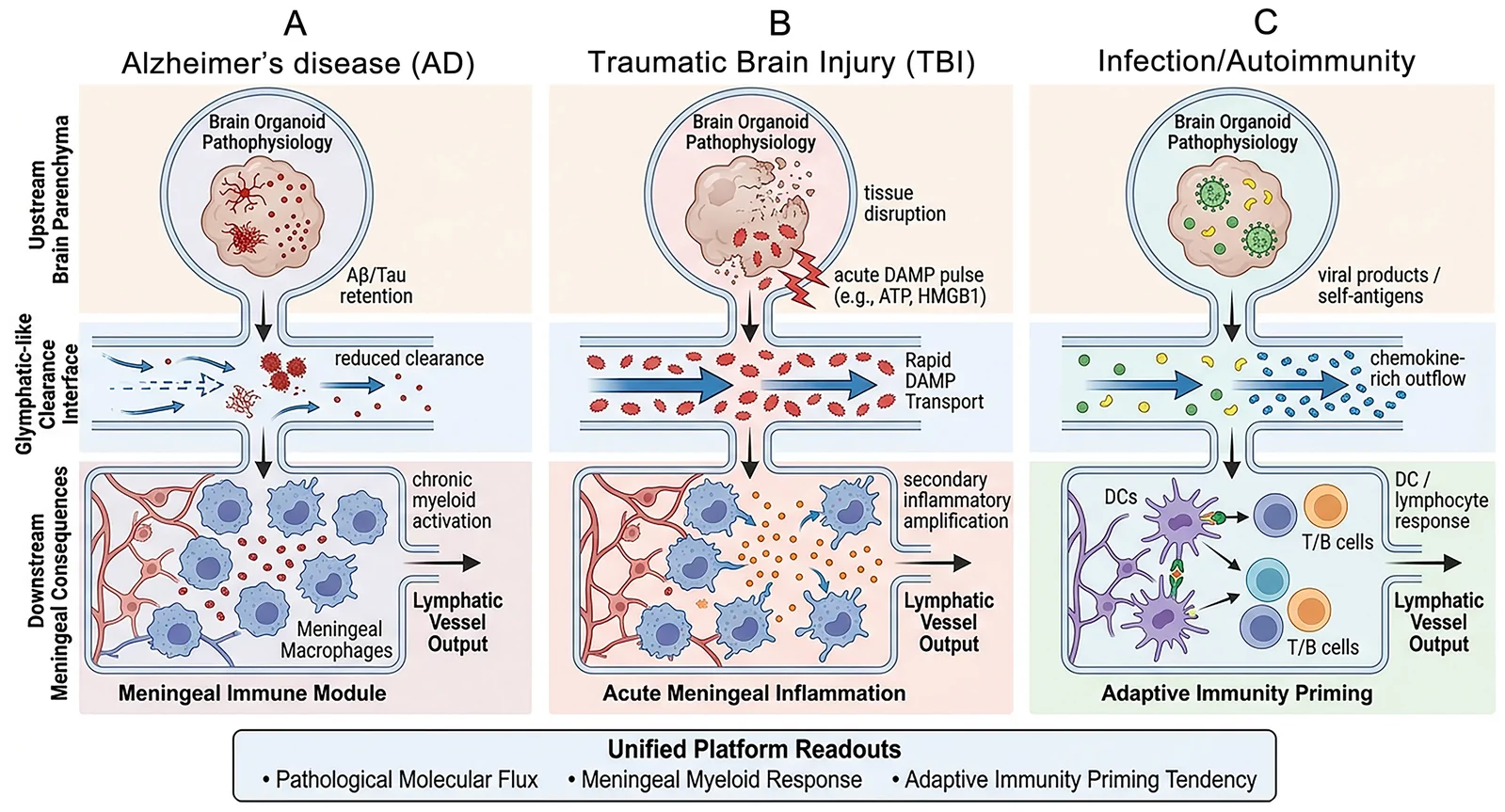
Application of integrated platforms in neurological diseases: from clearing obstacles to meningeal immune imbalance. This figure summarizes how the platform integrating brain organoids, lymphoid interfaces, and meningeal immune modules adapts in different disease scenarios. The left panel for Alzheimer’s disease (AD) **(A)** shows that Aβ and Tau accumulate in the brain parenchyma, while decreased AQP4 polarization and weakened lymphoid clearance lead to the retention of pathological proteins and prolonged low-level protein exposure at the meninges, ultimately inducing chronic myeloid cell activation. The intermediate panel for traumatic brain injury (TBI) **(B)** shows that mechanical tissue destruction rapidly induces the release of DAMPs such as adenosine triphosphate (ATP), high mobility group box 1 (HMGB1), S100 calcium-binding protein β (S100β), and neurofilament light chains, which quickly reach the meningeal module via the lymphoid interface, triggering acute borderline inflammation and amplifying secondary damage. The right panel for infection/autoimmunity **(C)** shows that virus-associated molecules, inflammatory chemokines, or neuronal autoantigens can enter the meningeal niche via a fluid system, driving local responses in dendritic cells, macrophages, and lymphocytes. The readout box at the bottom indicates that the platform can compare pathological molecular flux, meningeal myeloid response, and adaptive immune initiation tendency in different disease backgrounds within a unified system.

After truly integrating the brain parenchyma module, the lymphoid interface, and the meningeal lymphoid-immune module, this platform becomes a humanized system that can simulate the continuous process of pathological signal generation, fluid-mediated transport, and meningeal immune feedback (**Figure 6**). Its most important advantage is that it can simultaneously observe the causal relationship between brain parenchyma load, fluid clearance efficiency, and meningeal immune response in the same experimental platform (24, 72, 109).

In AD, Aβ and Tau not only accumulate in the brain, but their clearance efficiency and the way they reach the brain boundary also determine disease progression (110, 111). In this platform, pathological output can be established using iPSC-derived brain organoids with APP/PS1 mutations or APOE4 background, and the flux changes of pathological proteins can be analyzed by altering AQP4 polarization, flow rate, or choroid plexus-like input (31, 111, 112). If the Aβ/Tau flux in the effluent decreases while the meningeal module is exposed to low-level, persistent pathological stimulation for a long period, it is expected to more realistically simulate the chronic meningeal inflammation state in AD; conversely, if enhanced AQP4 polarization or meningeal lymphatic drainage leads to a decrease in pathological retention and a relief of meningeal inflammation, it will support the mechanism of clearance barrier-boundary inflammation coupling (25). The TBI model is better suited for studying how acute pathological signals are rapidly amplified at the brain boundary. By applying mechanical stretching, compression, microneedle injury, or high-intensity ultrasound injury to brain organoids, the release of DAMPs such as ATP, HMGB1, S100β, and neurofilament light chains can be induced, and whether these molecules rapidly reach the meningeal module via lymphoid pathways can be tracked (41, 113, 114). If mild injury itself does not cause severe meningeal inflammation, but the meningeal response is significantly enhanced under conditions of AQP4 misalignment or fluid outflow obstruction, this platform could be used to test whether the extension of secondary injury largely depends on the imbalance of brain boundary clearance, rather than solely on the degree of primary mechanical injury (115). In neuroinfection models, SARS-CoV-2-related proteins, viral RNA mimics, HIV-related proteins, or inflammatory factors can be introduced via the choroid plexus or brain parenchyma modules to observe whether these signals continuously expose the meningeal immune niche via a fluid system and induce CCL2, CXCL10, IL-6, or IFN-related responses (23, 42). In autoimmune-related diseases, this platform helps answer how brain-derived antigens are sampled by the boundary system and transformed into adaptive immune events. By inducing neuronal damage or increasing the release of N-methyl-D-aspartate receptor fragments, myelin antigens, etc., it is possible to observe whether these antigens enter the efflux end and are taken up by meningeal dendritic cells, thereby assessing their activation potential on T or B cells (9, 116–119).

At the translational level, this integrated platform is also well-suited for drug screening and patient stratification. It can simultaneously test different drug classes that promote brain parenchymal clearance, protect meningeal lymphoid structures, and suppress borderline inflammation, and observe the combined effects of these drugs on upstream pathological output, midstream fluid flux, and downstream meningeal immunity (24, 72). Combined with patient-derived iPSCs and immune cells, this platform also holds promise for establishing personalized disease models based on clearance-borderline immune response phenotypes, providing higher-resolution experimental evidence for precision medicine (24, 120).

## 6 Challenges, validation standards, and future closed loop platforms

**Figure 7:**
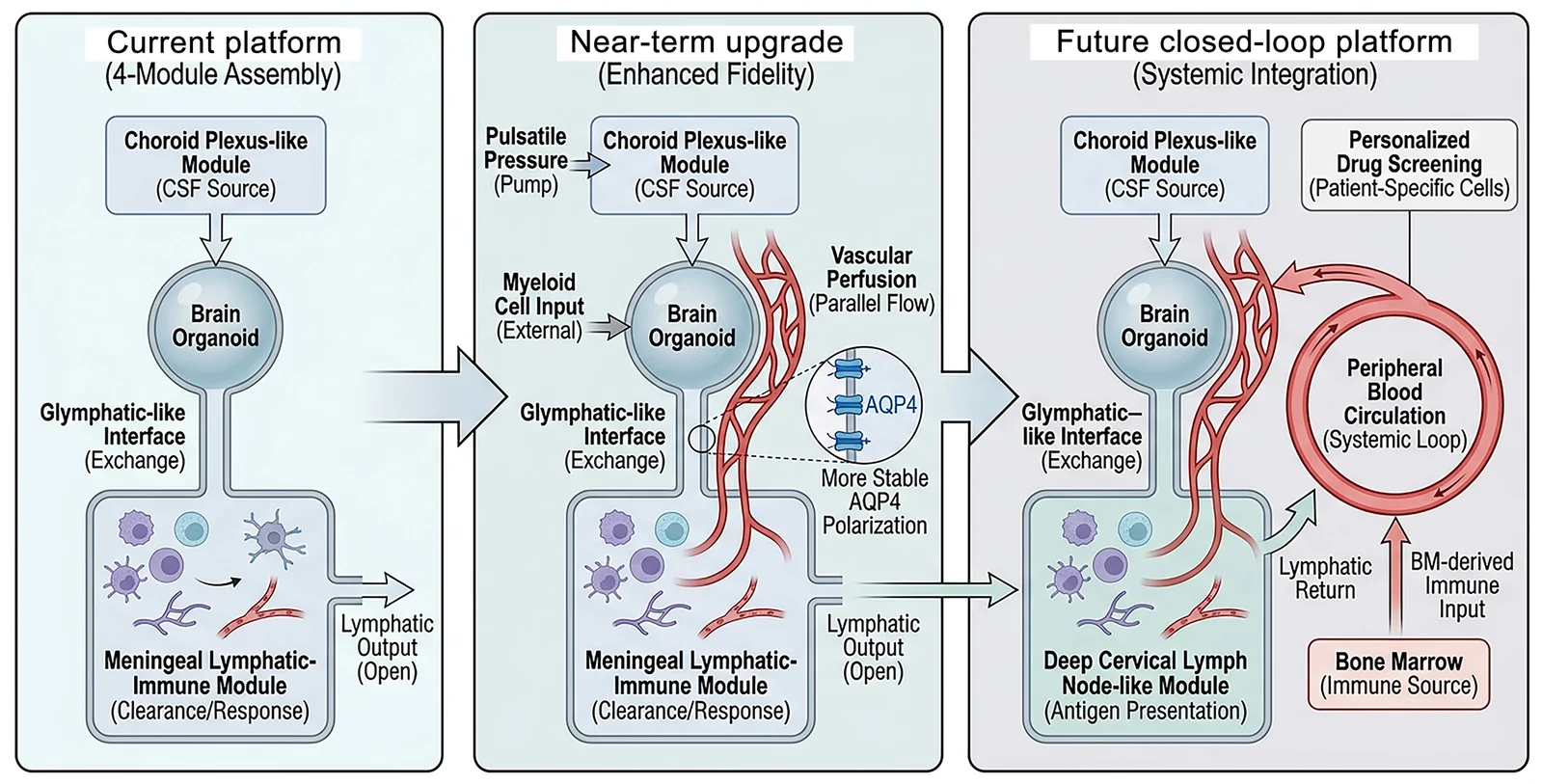
Development roadmap from four module assembled chip to closed loop humanized neuroimmune clearance platform. This diagram illustrates the current platform’s phased development framework. The left side shows the core four-module system proposed in this review, including choroid plexus-like CSF source, brain organoids, microfluidic exchange interfaces, and a meningeal lymphatic immune module. The middle, recent upgrade phase adds key elements closer to physiological states, such as vascular perfusion, pulsatile pressure, myeloid cell input, and a more stable astrocyte polarization interface, to improve cross-module fidelity and reproducibility. The right side represents the future closed-loop platform, further integrating deep cervical lymph node-like structures, peripheral blood circulation, bone marrow-derived immune input, and patient-specific cell sources, thus forming a truly closed-loop humanized neuroimmune clearance system. This diagram emphasizes that the future development goal is not to infinitely add complexity, but rather to gradually incorporate key modules that can significantly improve mechanism analysis and drug screening capabilities while maintaining interpretability and reproducibility. From an engineering perspective, the left-hand stage (available technologies/minimum viable product) represents the minimum viable research product that can be assembled using existing organoids, microfluidics, hydrogels, and analytical techniques. The middle stage (substantial optimization required) contains components requiring extensive optimization, including pulsatile flow, stable AQP4 polarization, vascular perfusion, and cross-modal culture compatibility. The right-hand stage (*de novo* development/closed-loop platform) contains functions that require *de novo* or near-*de novo* development, particularly controlled choroid plexus fluid output, a perfusionable human meningeal lymphoid immune microenvironment, bone marrow and lymph node coupling, and closed-loop immune cell recirculation.

This integrated framework is still in the early stages of moving from conceptual integration to standardized validation (**Figure 7**). The component-level analysis above indicates that the main bottleneck is not the complete absence of relevant *in vitro* technologies, but rather their functional integration under incompatible biological and engineering conditions. Brain organoids, choroid plexus organoids, on-chip lymphatic systems, and lymphatic vessel chips have all demonstrated some of the functions required by the proposed platform. However, these functions are typically achieved under different culture media, extracellular matrix, maturation periods, oxygen requirements, flow rates, and endpoint definitions. Therefore, the primary and most pressing challenge is cross-module compatibility: maintaining the phenotypes of neurons, astrocytes, microglia, choroid plexus epithelial cells, lymphatic endothelial cells, fibroblasts, macrophages, and dendritic cells in shared or isolated circuits, while avoiding the optimization of one module that compromises the stability of another (35, 103, 121, 122); The second challenge is the fidelity of quantitative transport. An effective clearance model must account for the mass of each tracer or pathological product entering the system, remaining in the parenchymal matrix, being taken up or degraded by cells, entering the lymphatic system, and ultimately appearing in the effluent. Without this mass balance, it is impossible to distinguish whether a decrease in effluent concentration is due to matrix adsorption, cellular uptake, degradation, or a genuine fluid transport impediment. flow rates, pressure gradients, matrix hydraulic resistance, and device adsorption must be measured, rather than treated as background parameters; The third challenge is functional maturation at the relevant interfaces. Of particular importance are stable AQP4 polarization at the astrocyte terminale, controlled access to choroid plexus-derived fluid, and perfusionable meningeal lymphatic endothelium capable of directed solute uptake; The fourth challenge is the reconstruction of the meningeal immune microenvironment. Marker expression alone is insufficient, the module must translate specific brain-derived signals into reproducible antigen uptake, cytokine production, antigen presentation, or immune cell migration; The fifth challenge is system-level validation using orthogonal human benchmarks, including cerebrospinal fluid composition, tracer kinetics, transcriptomic or spatial tissue references, and clinically or radiologically derived transport parameters. The second major issue is the still limited biological fidelity. While the current platform can model the continuous relationship between brain parenchyma and meninges relatively well, it still lacks true blood circulation, skull bone marrow input, deep cervical lymph nodes, and a complete adaptive immune closed loop. Therefore, the current system may be more suitable for modeling local meningeal sensing and early inflammatory amplification, but less suitable for studying systemic T cell priming, B cell maturation, or long-term peripheral immune feedback. This limitation needs to be clearly defined, especially for autoimmune encephalitis, chronic infections, or tumor-related neuroimmunological problems. The third key challenge is functional validation and imaging analysis. Because the system simultaneously involves thick tissues, low-velocity fluids, cell contact, and spatiotemporal distribution, a single endpoint indicator is not convincing. In the future, a three-tiered framework of structural validation, functional validation, and cross-system correspondence must be established. Validation should be conducted at three levels. The first level, identity and structure, determines cellular composition, spatial organization, viable tissue thickness, AQP4 localization, and patent vascular or lymphatic lumens; The second level, baseline function, determines neural activity, low spontaneous immune activation, molecular size-dependent transport, mass balance, lymphatic uptake, and positive control responsiveness; The third level, perturbation-response fidelity, requires that upstream perturbations produce measurable transport changes, subsequently generating time-sequential downstream immune responses. The proposed pathway disruption or restoration should alter downstream responses without causing nonspecific loss of cell viability. Each module should meet predefined pass/fail criteria before cross-module coupling. This requires demonstrating not only that cells and structures grow correctly, but also that fluid exchange, antigen uptake, and immune activation are matched, and that a conceptual mapping is established with *in vivo* tracing, meningeal lymphatic blockade, MRI fluid studies (123), or clinical meningeal imaging data (124, 125). Correspondingly, light-sheet microscopy, 3D live-cell tracking, spatial omics, and high-throughput detection of effluent will become indispensable technical supports in this field.

A phased approach is more realistic than attempting to assemble all modules simultaneously. The first stage should establish a minimum viable circuit, including a defined artificial source of CSF, AQP4-polarized parenchymal or glial vascular units, a downstream lymphoid endothelial cell pool, and quantitative tracer mass balance. The second stage, after achieving stable flow and transport criteria, should involve replacing the artificial cerebrospinal fluid source with cells derived from the choroid plexus, and adding meningeal fibroblasts, macrophages, and dendritic cells. The third stage may incorporate blood circulation, infusion of myeloid cells derived from the skull and bone marrow, equivalent cells from deep cervical lymph nodes, and recirculation of adaptive immune cells. Progress between stages should depend on pre-defined functional benchmarks, rather than the number of cell types included. Therefore, the technological requirements can be categorized into three types. The first type comprises technologies that are readily applicable, including organoid culture, microfluidic channel construction, hydrogel embedding, low flow rate perfusion, fluorescent tracer measurement, live-cell imaging, and multiplex effluent analysis. The second type comprises technologies requiring further optimization, including long-term organoid perfusion, low-adsorption materials, shared or compartmentalized culture media, AQP4 polarization, lymphatic endothelial stability, bubble control, and synchronized module maturation. The third type consists of entirely new elements requiring development, including the controlled collection of choroid plexus organoid derived fluids, standardized organoid CSF interfaces, fully human perfusionable meningeal lymphocyte matrix immune constructs, and validated system level quality balance frameworks. In the long term, the development direction of this field should be to build a truly closed-loop humanized neuroimmune clearance platform. Such a system should not only include choroid plexus, brain organoids, lymphoid fluid interfaces, and meningeal lymphatic immune modules, but should also further incorporate blood circulation, skull bone marrow-like myeloid input, and lymph node-like adaptive immune output (120, 126, 127). Future development should not focus on indiscriminately adding complexity, but rather on gradually adding modules that truly improve the explanatory and predictive capabilities of mechanisms, centered around the most critical biological questions.

## 7 Conclusion and outlook

Research on the meningeal lymphatic system, glymphatic-like CSF-ISF exchange, and the immune niche at the brain boundary is continuously driving the study of neurological diseases from localized lesions of the brain parenchyma to the research field of continuous imbalance between the brain parenchyma and the boundary (128). Increasing evidence suggests that key pathological events in AD (129–131), TBI (132, 133), neuroinfection (134), and autoimmune encephalitis (135) are not limited to neurons and glial cells themselves, but occur in a continuous process of pathological signal generation, boundary fluid transport, and meningeal immune sensing. We believe that integrating brain organoids, choroid plexus-like CSF sources, lymphoid microfluidic interfaces, and meningeal lymphoid-immune niches into a humanized assembled microarray platform holds promise for providing a new experimental framework for elucidating this continuous process. However, it is worth noting that the proposed assembled chip is still a conceptual integration framework, rather than an experimentally validated end to end platform. Its feasibility is supported by a multi-component model, while its predictive value will depend on demonstrating its cross- module compatibility, quantitative transfer conservatism, and consistency with human data. Currently available organoid and microphysiological system technologies are sufficient to build simplified, modular research prototypes, but not enough to achieve fully closed-loop products. Decisive progress depends less on increasing the number of cell types integrated and more on developing reproducible biological interfaces, quantitative quality balance, and human relevant validation standards. The significance of this integrated platform lies not in mechanically replicating all *in vivo* structures, but in reconstructing the causal chain of pathological signals-from their output in the brain parenchyma, through fluid systems, and ultimately sampled and amplified by the immune system at the meningeal boundary in a controllable, visualized, and quantifiable manner (109, 136). With the further integration of microfluidics, bioprinting, spatial omics, and human immune modeling technologies, a truly closed-loop humanized neuroimmune clearance platform is expected to gradually become a reality. Once such a system matures, it will not only deepen our understanding of brain boundary pathology but also provide new technological support for the study of the mechanisms of neurological diseases, drug development, and personalized precision treatment.

## References

[1] Louveau A Smirnov I Keyes TJ Eccles JD Rouhani SJ Peske JD . Structural and functional features of central nervous system lymphatic vessels. Nature. (2015) 523:337–41. doi: 10.1038/nature14432 26030524 PMC4506234

[2] Aspelund A Antila S Proulx ST Karlsen TV Karaman S Detmar M . A dural lymphatic vascular system that drains brain interstitial fluid and macromolecules. J Exp Med. (2015) 212:991–9. doi: 10.1084/jem.20142290 26077718 PMC4493418

[3] Ahn JH Cho H Kim JH Kim SH Ham JS Park I . Meningeal lymphatic vessels at the skull base drain cerebrospinal fluid. Nature. (2019) 572:62–6. doi: 10.1038/s41586-019-1419-5 31341278

[4] Plog BA Nedergaard M . The glymphatic system in central nervous system health and disease: past, present, and future. Annu Rev Pathol. (2018) 13:379–94. doi: 10.1146/annurev-pathol-051217-111018 29195051 PMC5803388

[5] Rustenhoven J Kipnis J . Brain borders at the central stage of neuroimmunology. Nature. (2022) 612:417–29. doi: 10.1038/s41586-022-05474-7 36517712 PMC10205171

[6] Iliff JJ Wang M Liao Y Plogg BA Peng W Gundersen GA . A paravascular pathway facilitates csf flow through the brain parenchyma and the clearance of interstitial solutes, including amyloid beta. Sci Transl Med. (2012) 4:147ra11. doi: 10.1126/scitranslmed.3003748 22896675 PMC3551275

[7] Rasmussen MK Mestre H Nedergaard M . The glymphatic pathway in neurological disorders. Lancet Neurol. (2018) 17:1016–24. doi: 10.1016/S1474-4422(18)30318-1 30353860 PMC6261373

[8] Mestre H Tithof J Du T Song W Peng W Sweeney AM . Flow of cerebrospinal fluid is driven by arterial pulsations and is reduced in hypertension. Nat Commun. (2018) 9:4878. doi: 10.1038/s41467-018-07318-3 30451853 PMC6242982

[9] Merlini A Haberl M Strauss J Hildebrand L Genc N Franz J . Distinct roles of the meningeal layers in cns autoimmunity. Nat Neurosci. (2022) 25:887–99. doi: 10.1038/s41593-022-01108-3 35773544

[10] Betsholtz C Engelhardt B Koh GY McDonald DM Proulx ST Siegenthaler J . Advances and controversies in meningeal biology. Nat Neurosci. (2024) 27:2056–72. doi: 10.1038/s41593-024-01701-8 39333784 PMC11862877

[11] Da Mesquita S Louveau A Vaccari A Smirnov I Cornelison RC Kingsmore KM . Functional aspects of meningeal lymphatics in ageing and Alzheimer's disease. Nature. (2018) 560:185–91. doi: 10.1038/s41586-018-0368-8 30046111 PMC6085146

[12] Plog BA Dashnaw ML Hitomi E Peng W Liao Y Lou N . Biomarkers of traumatic injury are transported from brain to blood via the glymphatic system. J Neurosci. (2015) 35:518–26. doi: 10.1523/JNEUROSCI.3742-14.2015 25589747 PMC4293408

[13] Jessen NA Munk AS Lundgaard I Nedergaard M . The glymphatic system: a beginner's guide. Neurochem Res. (2015) 40:2583–99. doi: 10.1007/s11064-015-1581-6 25947369 PMC4636982

[14] Peters ME Lyketsos CG . The glymphatic system's role in traumatic brain injury-related neurodegeneration. Mol Psychiatry. (2023) 28:2707–15. doi: 10.1038/s41380-023-02070-7 37185960

[15] Hsu M Laaker C Madrid A Herbath M Choi YH Sandor M . Neuroinflammation creates an immune regulatory niche at the meningeal lymphatic vasculature near the cribriform plate. Nat Immunol. (2022) 23:581–93. doi: 10.1038/s41590-022-01158-6 35347285 PMC8989656

[16] Klein RS Garber C Howard N . Infectious immunity in the central nervous system and brain function. Nat Immunol. (2017) 18:132–41. doi: 10.1038/ni.3656 28092376 PMC5815515

[17] Sloan SA Andersen J Pasca AM Birey F Pasca SP . Generation and assembly of human brain region-specific three-dimensional cultures. Nat Protoc. (2018) 13:2062–85. doi: 10.1038/s41596-018-0032-7 30202107 PMC6597009

[18] Park DS Kozaki T Tiwari SK Moreira M Khalilnezhad A Torta F . Ips-cell-derived microglia promote brain organoid maturation via cholesterol transfer. Nature. (2023) 623:397–405. doi: 10.1038/s41586-023-06713-1 37914940

[19] Sun XY Ju XC Li Y Zeng PM Wu J Zhou YY . Generation of vascularized brain organoids to study neurovascular interactions. Elife. (2022) 11:e76707. doi: 10.7554/eLife.76707 35506651 PMC9246368

[20] Qian X Nguyen HN Song MM Hadiono C Ogden SC Hammack C . Brain-region-specific organoids using mini-bioreactors for modeling zikv exposure. Cell. (2016) 165:1238–54. doi: 10.1016/j.cell.2016.04.032 27118425 PMC4900885

[21] Velasco S Kedaigle AJ Simmons SK Nash A Rocha M Quadrato G . Individual brain organoids reproducibly form cell diversity of the human cerebral cortex. Nature. (2019) 570:523–7. doi: 10.1038/s41586-019-1289-x 31168097 PMC6906116

[22] Pellegrini L Bonfio C Chadwick J Begum F Skehel M Lancaster MA . Human cns barrier-forming organoids with cerebrospinal fluid production. Science. (2020) 369(6500):eaaz5626. doi: 10.1126/science.aaz5626 32527923 PMC7116154

[23] Jacob F Pather SR Huang WK Zhang F Wong SZH Zhou H . Human pluripotent stem cell-derived neural cells and brain organoids reveal sars-cov-2 neurotropism predominates in choroid plexus epithelium. Cell Stem Cell. (2020) 27:937–50:e9. doi: 10.1016/j.stem.2020.09.016 33010822 PMC7505550

[24] Ingber DE . Human organs-on-chips for disease modelling, drug development and personalized medicine. Nat Rev Genet. (2022) 23:467–91. doi: 10.1038/s41576-022-00466-9 35338360 PMC8951665

[25] Soden PA Henderson AR Lee E . A microfluidic model of aqp4 polarization dynamics and fluid transport in the healthy and inflamed human brain: the first step towards glymphatics-on-a-chip. Adv Biol (Weinh). (2022) 6:e2200027. doi: 10.1002/adbi.202200027 35922370 PMC9771879

[26] Yslas AR Park R Nishimura N Lee E . Monomeric and oligomeric amyloid-beta cause distinct Alzheimer's disease pathophysiological characteristics in astrocytes in human glymphatics-on-chip models. Lab Chip. (2024) 24:3826–39. doi: 10.1039/d4lc00287c 39037244 PMC11302770

[27] Jalilian E Shin SR . Novel model of cortical-meningeal organoid co-culture system improves human cortical brain organoid cytoarchitecture. Sci Rep. (2023) 13:7809. doi: 10.1038/s41598-023-35077-9 37183210 PMC10183460

[28] Miura Y Li MY Revah O Yoon SJ Narazaki G Pasca SP . Engineering brain assembloids to interrogate human neural circuits. Nat Protoc. (2022) 17:15–35. doi: 10.1038/s41596-021-00632-z 34992269

[29] Cakir B Xiang Y Tanaka Y Kural MH Parent M Kang YJ . Engineering of human brain organoids with a functional vascular-like system. Nat Methods. (2019) 16:1169–75. doi: 10.1038/s41592-019-0586-5 31591580 PMC6918722

[30] Lange S Ebeling M Loye A Wanke F Siebourg-Polster J Sudharshan TJJ . Human myelinated brain organoids with integrated microglia as a model for myelin repair and remyelinating therapies. Sci Transl Med. (2025) 17:eadp7047. doi: 10.1126/scitranslmed.adp7047 40929244

[31] Lin YT Seo J Gao F Feldman HM Wen HL Penney J . Apoe4 causes widespread molecular and cellular alterations associated with Alzheimer's disease phenotypes in human ipsc-derived brain cell types. Neuron. (2018) 98:1141–54:e7. doi: 10.1016/j.neuron.2018.05.008 29861287 PMC6023751

[32] Chen X Sun G Tian E Zhang M Davtyan H Beach TG . Modeling sporadic Alzheimer's disease in human brain organoids under serum exposure. Adv Sci (Weinh). (2021) 8:e2101462. doi: 10.1002/advs.202101462 34337898 PMC8456220

[33] Hongxi W Ruting W Yiyang L Qinglian H Jibing C Feng J . Human brain organoids: development and applications. J Microbiol Biotechnol. (2025) 35:e2411040. doi: 10.4014/jmb.2411.11040 40443221 PMC12149405

[34] Stanton AE Bubnys A Agbas E James B Park DS Jiang A . Engineered 3d immuno-glial-neurovascular human mibrain model. Proc Natl Acad Sci USA. (2025) 122:e2511596122. doi: 10.1073/pnas.2511596122 41105712 PMC12557797

[35] Lancaster MA Renner M Martin CA Wenzel D Bicknell LS Hurles ME . Cerebral organoids model human brain development and microcephaly. Nature. (2013) 501:373–9. doi: 10.1038/nature12517 23995685 PMC3817409

[36] Xie L Kang H Xu Q Chen MJ Liao Y Thiyagarajan M . Sleep drives metabolite clearance from the adult brain. Science. (2013) 342:373–7. doi: 10.1126/science.1241224 24136970 PMC3880190

[37] Eidsvaag VA Enger R Hansson HA Eide PK Nagelhus EA . Human and mouse cortical astrocytes differ in aquaporin-4 polarization toward microvessels. Glia. (2017) 65:964–73. doi: 10.1002/glia.23138 28317216 PMC5413834

[38] Abud EM Ramirez RN Martinez ES Healy LM Nguyen CHH Newman SA . Ipsc-derived human microglia-like cells to study neurological diseases. Neuron. (2017) 94:278–93:e9. doi: 10.1016/j.neuron.2017.03.042 28426964 PMC5482419

[39] Cakir B Tanaka Y Kiral FR Xiang Y Dagliyan O Wang J . Expression of the transcription factor pu.1 induces the generation of microglia-like cells in human cortical organoids. Nat Commun. (2022) 13:430. doi: 10.1038/s41467-022-28043-y 35058453 PMC8776770

[40] Wu Y Cheng J Qi J Hang C Dong R Low BC . Three-dimensional liquid metal-based neuro-interfaces for human hippocampal organoids. Nat Commun. (2024) 15:4047. doi: 10.1038/s41467-024-48452-5 38744873 PMC11094048

[41] Lai JD Berlind JE Fricklas G Lie C Urenda JP Lam K . Kcnj2 inhibition mitigates mechanical injury in a human brain organoid model of traumatic brain injury. Cell Stem Cell. (2024) 31:519–36:e8. doi: 10.1016/j.stem.2024.03.004 38579683

[42] Samudyata Oliveira AO Malwade S Rufino de Sousa N Goparaju SK Gracias J . Sars-cov-2 promotes microglial synapse elimination in human brain organoids. Mol Psychiatry. (2022) 27:3939–50. doi: 10.1038/s41380-022-01786-2 36198765 PMC9533278

[43] Pasca SP . The rise of three-dimensional human brain cultures. Nature. (2018) 553:437–45. doi: 10.1038/nature25032 29364288

[44] Cho AN Jin Y An Y Kim J Choi YS Lee JS . Microfluidic device with brain extracellular matrix promotes structural and functional maturation of human brain organoids. Nat Commun. (2021) 12:4730. doi: 10.1038/s41467-021-24775-5 34354063 PMC8342542

[45] Kang R Park S Shin S Bak G Park JC . Electrophysiological insights with brain organoid models: a brief review. BMB Rep. (2024) 57:311–7. doi: 10.5483/BMBRep.2024-0077 38919012 PMC11289503

[46] Bonner MG Gudapati H Mou X Musah S . Microfluidic systems for modeling human development. Development. (2022) 149(3):dev199463. doi: 10.1242/dev.199463 35156682 PMC8918817

[47] Kettunen P Ruuska J Quirin T Ojha R Saber SH Sng JDJ . Sars-cov-2 infection in hipsc-derived neurons is cathepsin-dependent and causes differential accumulation of hif1a and phosphorylated tau. Mol Ther Nucleic Acids. (2025) 36:102726. doi: 10.1016/j.omtn.2025.102726 41216428 PMC12597281

[48] Fedorchak NJ Iyer N Ashton RS . Bioengineering tissue morphogenesis and function in human neural organoids. Semin Cell Dev Biol. (2021) 111:52–9. doi: 10.1016/j.semcdb.2020.05.025 32540123 PMC8755418

[49] Giandomenico SL Mierau SB Gibbons GM Wenger LMD Masullo L Sit T . Cerebral organoids at the air-liquid interface generate diverse nerve tracts with functional output. Nat Neurosci. (2019) 22:669–79. doi: 10.1038/s41593-019-0350-2 30886407 PMC6436729

[50] Quadrato G Nguyen T Macosko EZ Sherwood JL Min Yang S Berger DR . Cell diversity and network dynamics in photosensitive human brain organoids. Nature. (2017) 545:48–53. doi: 10.1038/nature22047 28445462 PMC5659341

[51] Lancaster MA Knoblich JA . Generation of cerebral organoids from human pluripotent stem cells. Nat Protoc. (2014) 9:2329–40. doi: 10.1038/nprot.2014.158 25188634 PMC4160653

[52] Ormel PR Vieira de Sa R van Bodegraven EJ Karst H Harschnitz O Sneeboer MAM . Microglia innately develop within cerebral organoids. Nat Commun. (2018) 9:4167. doi: 10.1038/s41467-018-06684-2 30301888 PMC6177485

[53] Muffat J Li Y Yuan B Mitalipova M Omer A Corcoran S . Efficient derivation of microglia-like cells from human pluripotent stem cells. Nat Med. (2016) 22:1358–67. doi: 10.1038/nm.4189 27668937 PMC5101156

[54] Haenseler W Sansom SN Buchrieser J Newey SE Moore CS Nicholls FJ . A highly efficient human pluripotent stem cell microglia model displays a neuronal-co-culture-specific expression profile and inflammatory response. Stem Cell Rep. (2017) 8:1727–42. doi: 10.1016/j.stemcr.2017.05.017 28591653 PMC5470330

[55] Xu R Boreland AJ Li X Erickson C Jin M Atkins C . Developing human pluripotent stem cell-based cerebral organoids with a controllable microglia ratio for modeling brain development and pathology. Stem Cell Rep. (2021) 16:1923–37. doi: 10.1016/j.stemcr.2021.06.011 34297942 PMC8365109

[56] Frost JL Schafer DP . Microglia: architects of the developing nervous system. Trends Cell Biol. (2016) 26:587–97. doi: 10.1016/j.tcb.2016.02.006 27004698 PMC4961529

[57] Bennett ML Bennett FC Liddelow SA Ajami B Zamanian JL Fernhoff NB . New tools for studying microglia in the mouse and human CNS. Proc Natl Acad Sci USA. (2016) 113:E1738–46. doi: 10.1073/pnas.1525528113 26884166 PMC4812770

[58] Haynes SE Hollopeter G Yang G Kurpius D Dailey ME Gan WB . The P2Y12 receptor regulates microglial activation by extracellular nucleotides. Nat Neurosci. (2006) 9:1512–9. doi: 10.1038/nn1805 17115040

[59] Krasemann S Madore C Cialic R Baufeld C Calcagno N El Fatimy R . The TREM2-APOE pathway drives the transcriptional phenotype of dysfunctional microglia in neurodegenerative diseases. Immunity. (2017) 47:566–81:e9. doi: 10.1016/j.immuni.2017.08.008 28930663 PMC5719893

[60] Wu LJ Vadakkan KI Zhuo M . ATP-induced chemotaxis of microglial processes requires P2Y receptor-activated initiation of outward potassium currents. Glia. (2007) 55:810–21. doi: 10.1002/glia.20500 17357150

[61] Karabova MK Del Ser-Badia A Hedegaard A Washer SJ Baykam Z O'Brien DP . Tracking tau and cellular responses in human iPSC-microglia: From uptake to seedable secretion, including in extracellular vesicles. Alzheimers Dement. (2026) 22:e71337. doi: 10.1002/alz.71337 41943483 PMC13053939

[62] Jacquet RG Gonzalez Ibanez F Picard K Funes L Khakpour M Gouras GK . Microglia degrade Alzheimer's amyloid-beta deposits extracellularly via digestive exophagy. Cell Rep. (2024) 43:115052. doi: 10.1016/j.celrep.2024.115052 39644493 PMC11760508

[63] Lv Z Chen L Chen P Peng H Rong Y Hong W . Clearance of beta-amyloid and synapses by the optogenetic depolarization of microglia is complement selective. Neuron. (2024) 112:740–54:e7. doi: 10.1016/j.neuron.2023.12.003 38295790

[64] Heneka MT Kummer MP Stutz A Delekate A Schwartz S Vieira-Saecker A . NLRP3 is activated in Alzheimer's disease and contributes to pathology in APP/PS1 mice. Nature. (2013) 493:674–8. doi: 10.1038/nature11729 23254930 PMC3812809

[65] Banerjee P Paza E Perkins EM James OG Kenkhuis B Lloyd AF . Generation of pure monocultures of human microglia-like cells from induced pluripotent stem cells. Stem Cell Res. (2020) 49:102046. doi: 10.1016/j.scr.2020.102046 33096385

[66] Martinez-Meza S Premeaux TA Cirigliano SM Friday CM Michael S Mediouni S . Antiretroviral drug therapy does not reduce neuroinflammation in an HIV-1 infection brain organoid model. J Neuroinflamm. (2025) 22(1):66. doi: 10.1186/s12974-025-03375-w 40045391 PMC11881274

[67] Steffen JF Widerspick L Jansen S Tappe D . A human cerebral organoid model of West Nile virus encephalitis shows innate immunocompetency. Nat Commun. (2026) 17. doi: 10.1038/s41467-026-70281-x 41794851 PMC12976376

[68] Venegas C Kumar S Franklin BS Dierkes T Brinkschulte R Tejera D . Microglia-derived ASC specks cross-seed amyloid-beta in Alzheimer's disease. Nature. (2017) 552:355–61. doi: 10.1038/nature25158 29293211

[69] Spitz S Ko E Ertl P Kamm RD . How organ-on-a-chip technology can assist in studying the role of the glymphatic system in neurodegenerative diseases. Int J Mol Sci. (2023) 24(3):2171. doi: 10.3390/ijms24032171 36768495 PMC9916687

[70] Liu Q Yan L Huang M Zeng H Satyanarayanan SK Shi Z . Experimental alcoholism primes structural and functional impairment of the glymphatic pathway. Brain Behav Immun. (2020) 85:106–19. doi: 10.1016/j.bbi.2019.06.029 31247290

[71] Kitchen P Salman MM Halsey AM Clarke-Bland C MacDonald JA Ishida H . Targeting aquaporin-4 subcellular localization to treat central nervous system edema. Cell. (2020) 181:784–99:e19. doi: 10.1016/j.cell.2020.03.037 32413299 PMC7242911

[72] Low LA Mummery C Berridge BR Austin CP Tagle DA . Organs-on-chips: Into the next decade. Nat Rev Drug Discov. (2021) 20:345–61. doi: 10.1038/s41573-020-0079-3 32913334

[73] Ilan IS Yslas AR Peng Y Lu R Lee E . A 3D human lymphatic vessel-on-chip reveals the roles of interstitial flow and VEGF-A/C for lymphatic sprouting and discontinuous junction formation. Cell Mol Bioeng. (2023) 16:325–39. doi: 10.1007/s12195-023-00780-0 37811004 PMC10550886

[74] Sakka L Coll G Chazal J . Anatomy and physiology of cerebrospinal fluid. Eur Ann Otorhinolaryngol Head Neck Dis. (2011) 128:309–16. doi: 10.1016/j.anorl.2011.03.002 22100360

[75] Tariq K Toma A Khawari S Amarouche M Elborady MA Thorne L . Cerebrospinal fluid production rate in various pathological conditions: A preliminary study. Acta Neurochir (Wien). (2023) 165:2309–19. doi: 10.1007/s00701-023-05650-2 37354286 PMC10409822

[76] DeStefano JG Xu ZS Williams AJ Yimam N Searson PC . Effect of shear stress on iPSC-derived human brain microvascular endothelial cells (DHBMECs). Fluids Barriers CNS. (2017) 14:20. doi: 10.1186/s12987-017-0068-z 28774343 PMC5543552

[77] Muniz AJ Topal T Brooks MD Sze A Kim DH Jordahl J . Engineered extracellular matrices facilitate brain organoids from human pluripotent stem cells. Ann Clin Transl Neurol. (2023) 10:1239–53. doi: 10.1002/acn3.51820 37283238 PMC10351667

[78] Campbell SB Wu Q Yazbeck J Liu C Okhovatian S Radisic M . Beyond polydimethylsiloxane: Alternative materials for fabrication of organ-on-a-chip devices and microphysiological systems. ACS Biomater Sci Eng. (2021) 7:2880–99. doi: 10.1021/acsbiomaterials.0c00640 34275293

[79] van Midwoud PM Janse A Merema MT Groothuis GM Verpoorte E . Comparison of biocompatibility and adsorption properties of different plastics for advanced microfluidic cell and tissue culture models. Anal Chem. (2012) 84:3938–44. doi: 10.1021/ac300771z 22444457

[80] Feutry F Simon N Genay S Lannoy D Barthelemy C Decaudin B . Stability of 10 mg/ml cefuroxime solution for intracameral injection in commonly used polypropylene syringes and new ready-to-use cyclic olefin copolymer sterile vials using the LC-UV stability-indicating method. Drug Dev Ind Pharm. (2016) 42:166–74. doi: 10.3109/03639045.2015.1038273 26006333

[81] Kim S Min S Choi YS Jo SH Jung JH Han K . Tissue extracellular matrix hydrogels as alternatives to Matrigel for culturing gastrointestinal organoids. Nat Commun. (2022) 13:1692. doi: 10.1038/s41467-022-29279-4 35354790 PMC8967832

[82] Schwerk C Schroten H . *In vitro* models of the choroid plexus and the blood-cerebrospinal fluid barrier: Advances, applications, and perspectives. Hum Cell. (2024) 37:1235–42. doi: 10.1007/s13577-024-01115-5 39103559 PMC11341628

[83] Harding AT Gehrke L Vyas JM Harding HB . Human brain organoids: A new model to study Cryptococcus neoformans neurotropism. J Fungi (Basel). (2025) 11(7):539. doi: 10.3390/jof11070539 40985448 PMC12295756

[84] Segeroth M Wachsmuth L Gagel M Albers F Hess A Faber C . Disentangling the impact of cerebrospinal fluid formation and neuronal activity on solute clearance from the brain. Fluids Barriers CNS. (2023) 20:43. doi: 10.1186/s12987-023-00443-2 37316849 PMC10265831

[85] Hebisch M Klostermeier S Wolf K Boccaccini AR Wolf SE Tanzi RE . The impact of the cellular environment and aging on modeling Alzheimer's disease in 3D cell culture models. Adv Sci (Weinh). (2023) 10:e2205037. doi: 10.1002/advs.202205037 36642841 PMC10015857

[86] Cugurra A Mamuladze T Rustenhoven J Dykstra T Beroshvili G Greenberg ZJ . Skull and vertebral bone marrow are myeloid cell reservoirs for the meninges and CNS parenchyma. Science. (2021) 373(6553):eabf7844. doi: 10.1126/science.abf7844 34083447 PMC8863069

[87] Alves de Lima K Rustenhoven J Da Mesquita S Wall M Salvador AF Smirnov I . Meningeal gammadelta T cells regulate anxiety-like behavior via IL-17A signaling in neurons. Nat Immunol. (2020) 21:1421–9. doi: 10.1038/s41590-020-0776-4 32929273 PMC8496952

[88] Louveau A Herz J Alme MN Salvador AF Dong MQ Viar KE . CNS lymphatic drainage and neuroinflammation are regulated by meningeal lymphatic vasculature. Nat Neurosci. (2018) 21:1380–9. doi: 10.1038/s41593-018-0227-9 30224810 PMC6214619

[89] Tamadon A Afshar A Mussin NM Zhilisbayeva KR Kurmanalina MA Baspakova A . Mouse brain lymphatic vessels. ACS Chem Neurosci. (2025) 16:4492–501. doi: 10.1021/acschemneuro.5c00533 41263424

[90] Biancheri D Rossini L Cagnoli C De Santis D Parente A Ferrario R . Corpora amylacea and glymphatic system in temporal lobe epilepsy patients: Clinicopathological correlation. Epilepsia. (2026). doi: 10.1002/epi.70285 42132920 PMC13525605

[91] Li MN Jing YH Wu C Li X Liang FY Li G . Continuous theta burst stimulation dilates meningeal lymphatic vessels by up-regulating VEGF-C in meninges. Neurosci Lett. (2020) 735:135197. doi: 10.1016/j.neulet.2020.135197 32590044

[92] Roberts LM Hammel JH Jin P Cunningham JJ Schumaecker S Davis S . Demonstration of chemotherapeutic-mediated changes in meningeal lymphatics *in vitro*, ex vivo, and *in vivo*. Commun Biol. (2025) 8:1378. doi: 10.1038/s42003-025-08784-4 41083724 PMC12518801

[93] Song E Mao T Dong H Boisserand LSB Antila S Bosenberg M . VEGF-C-driven lymphatic drainage enables immunosurveillance of brain tumours. Nature. (2020) 577:689–94. doi: 10.1038/s41586-019-1912-x 31942068 PMC7100608

[94] Boisserand LSB Geraldo LH Bouchart J El Kamouh MR Lee S Sanganahalli BG . VEGF-C prophylaxis favors lymphatic drainage and modulates neuroinflammation in a stroke model. J Exp Med. (2024) 221(4):e20221983. doi: 10.1084/jem.20221983 38442272 PMC10913814

[95] Da Mesquita S Fu Z Kipnis J . The meningeal lymphatic system: A new player in neurophysiology. Neuron. (2018) 100:375–88. doi: 10.1016/j.neuron.2018.09.022 30359603 PMC6268162

[96] Antila S Karaman S Nurmi H Airavaara M Voutilainen MH Mathivet T . Development and plasticity of meningeal lymphatic vessels. J Exp Med. (2017) 214:3645–67. doi: 10.1084/jem.20170391 29141865 PMC5716035

[97] Jones HE Robertson GL Bodnya C Romero-Morales A O'Rourke R Gama V . Leptomeningeal neural organoid fusions as models to study meninges-brain signaling. Stem Cells Dev. (2025) 34:152–63. doi: 10.1089/scd.2024.0231 40126161 PMC12021768

[98] Kalaga P Ray SK . Organoids to model tumor microenvironment in progression of pathogenesis and treatment resistance in glioblastoma multiforme. Brain Sci. (2026) 16(5):531. doi: 10.3390/brainsci16050531 42192843 PMC13204722

[99] Schain AJ Melo-Carrillo A Borsook D Grutzendler J Strassman AM Burstein R . Activation of pial and dural macrophages and dendritic cells by cortical spreading depression. Ann Neurol. (2018) 83:508–21. doi: 10.1002/ana.25169 29394508 PMC5965700

[100] Liu Z Huang Y Wang X Li JY Zhang C Yang Y . The cervical lymph node contributes to peripheral inflammation related to Parkinson's disease. J Neuroinflamm. (2023) 20:93. doi: 10.1186/s12974-023-02770-5 37038192 PMC10088204

[101] Cheng X Li Y Wang H . Activation of wnt/beta-catenin signal induces dcs to differentiate into immune tolerant regdcs in septic mice. Mol Immunol. (2024) 172:38–46. doi: 10.1016/j.molimm.2024.04.015 38870636

[102] Korn T Kallies A . T cell responses in the central nervous system. Nat Rev Immunol. (2017) 17:179–94. doi: 10.1038/nri.2016.144 28138136

[103] Rustenhoven J Drieu A Mamuladze T de Lima KA Dykstra T Wall M . Functional characterization of the dural sinuses as a neuroimmune interface. Cell. (2021) 184:1000–16:e27. doi: 10.1016/j.cell.2020.12.040 33508229 PMC8487654

[104] Goyal G Prabhala P Mahajan G Bausk B Gilboa T Xie L . Ectopic lymphoid follicle formation and human seasonal influenza vaccination responses recapitulated in an organ-on-a-chip. Adv Sci (Weinh). (2022) 9:e2103241. doi: 10.1002/advs.202103241 35289122 PMC9109055

[105] Ewing-Crystal NA Mroz NM Larpthaveesarp A Lizama CO Pennington R Chiaranunt P . Dynamic fibroblast-immune interactions shape recovery after brain injury. Nature. (2025) 646:934–44. doi: 10.1038/s41586-025-09449-2 40903576 PMC12545229

[106] Santorella E Balsbaugh JL Ge S Saboori P Baker D Pachter JS . Proteomic interrogation of the meninges reveals the molecular identities of structural components and regional distinctions along the cns axis. Fluids Barriers CNS. (2023) 20:74. doi: 10.1186/s12987-023-00473-w 37858244 PMC10588166

[107] Guo X Zhu Y Gao S Wu Y Hong Y . Meningeal macrophages regulate fibroblasts to influence meningeal lymphatic function following traumatic brain injury. Theranostics. (2026) 16:5185–203. doi: 10.7150/thno.130263 41993613 PMC13080450

[108] DeSisto J O'Rourke R Jones HE Pawlikowski B Malek AD Bonney S . Single-cell transcriptomic analyses of the developing meninges reveal meningeal fibroblast diversity and function. Dev Cell. (2020) 54:43–59:e4. doi: 10.1016/j.devcel.2020.06.009 32634398 PMC7769050

[109] Andersson-Rolf A Clevers H . New developments and applications of human organoids. Nat Rev Mol Cell Biol. (2026) 27:543–58. doi: 10.1038/s41580-026-00974-0 42115752

[110] Roemer-Cassiano SN Wagner F Evangelista L Rauchmann BS Dehsarvi A Steward A . Amyloid-associated hyperconnectivity drives tau spread across connected brain regions in Alzheimer's disease. Sci Transl Med. (2025) 17:eadp2564. doi: 10.1126/scitranslmed.adp2564 39841807

[111] Zhang Z Li S Xu L Xiao S Li Y Huang J . Amyloid-beta-driven glymphatic dysfunction in Alzheimer's disease model mice is driven by Ca(2+)-mediated increases in astrocytic cholesterol. Nat Neurosci. (2026) 29:1369–85. doi: 10.1038/s41593-026-02261-9 41986732

[112] Xu X Yang X Zhang J Wang Y Selim M Zheng Y . Choroid plexus free-water correlates with glymphatic function in Alzheimer's disease. Alzheimers Dement. (2025) 21:e70239. doi: 10.1002/alz.70239 40394891 PMC12092370

[113] Li P Sun S Zhu X Liu X Yin R Chen Y . Intranasal delivery of engineered extracellular vesicles promotes neurofunctional recovery in traumatic brain injury. J Nanobiotechnol. (2025) 23:229. doi: 10.1186/s12951-025-03181-9 40114197 PMC11927228

[114] Manivannan S Marei O Elalfy O Zaben M . Neurogenesis after traumatic brain injury - the complex role of hmgb1 and neuroinflammation. Neuropharmacology. (2021) 183:108400. doi: 10.1016/j.neuropharm.2020.108400 33189765

[115] Candanedo C Doron O Hemphill JC Ramirez de Noriega F Manley GT Patal R . Characterizing the response to cerebrospinal fluid drainage in patients with an external ventricular drain: the pressure equalization ratio. Neurocrit Care. (2019) 30:340–7. doi: 10.1007/s12028-018-0612-y 30251075

[116] Dalmau J Lancaster E Martinez-Hernandez E Rosenfeld MR Balice-Gordon R . Clinical experience and laboratory investigations in patients with anti-nmdar encephalitis. Lancet Neurol. (2011) 10:63–74. doi: 10.1016/S1474-4422(10)70253-2 21163445 PMC3158385

[117] Gao W Kim MW Dykstra T Du S Boskovic P Lichti CF . Engineered t cell therapy for central nervous system injury. Nature. (2024) 634:693–701. doi: 10.1038/s41586-024-07906-y 39232158

[118] Jain RW Yong VW . B cells in central nervous system disease: diversity, locations and pathophysiology. Nat Rev Immunol. (2022) 22:513–24. doi: 10.1038/s41577-021-00652-6 34903877 PMC8667979

[119] Enose-Akahata Y Wang L Almsned F Johnson KR Mina Y Ohayon J . The repertoire of csf antiviral antibodies in patients with neuroinflammatory diseases. Sci Adv. (2023) 9:eabq6978. doi: 10.1126/sciadv.abq6978 36598996 PMC9812372

[120] Wang Q Yang Y Chen Z Li B Niu Y Li X . Lymph node-on-chip technology: cutting-edge advances in immune microenvironment simulation. Pharmaceutics. (2024) 16(5):666. doi: 10.3390/pharmaceutics16050666 38794327 PMC11124897

[121] Drager NM Sattler SM Huang CT Teter OM Leng K Hashemi SH . A crispri/a platform in human ipsc-derived microglia uncovers regulators of disease states. Nat Neurosci. (2022) 25:1149–62. doi: 10.1038/s41593-022-01131-4 35953545 PMC9448678

[122] Santos CRA Duarte AC Costa AR Tomas J Quintela T Goncalves I . The senses of the choroid plexus. Prog Neurobiol. (2019) 182:101680. doi: 10.1016/j.pneurobio.2019.101680 31404591

[123] Ringstad G Vatnehol SAS Eide PK . Glymphatic mri in idiopathic normal pressure hydrocephalus. Brain. (2017) 140:2691–705. doi: 10.1093/brain/awx191 28969373 PMC5841149

[124] Zhou Y Cai J Zhang W Gong X Yan S Zhang K . Impairment of the glymphatic pathway and putative meningeal lymphatic vessels in the aging human. Ann Neurol. (2020) 87:357–69. doi: 10.1002/ana.25670 31916277

[125] Lee DA Lee J Park KM . Glymphatic system impairment in patients with status epilepticus. Neuroradiology. (2022) 64:2335–42. doi: 10.1007/s00234-022-03018-4 35835880

[126] Deng S Li C Cao J Cui Z Du J Fu Z . Organ-on-a-chip meets artificial intelligence in drug evaluation. Theranostics. (2023) 13:4526–58. doi: 10.7150/thno.87266 37649608 PMC10465229

[127] Quintard C Tubbs E Jonsson G Jiao J Wang J Werschler N . A microfluidic platform integrating functional vascularized organoids-on-chip. Nat Commun. (2024) 15:1452. doi: 10.1038/s41467-024-45710-4 38365780 PMC10873332

[128] Zhang Q Niu Y Li Y Xia C Chen Z Chen Y . Meningeal lymphatic drainage: novel insights into central nervous system disease. Signal Transduct Target Ther. (2025) 10:142. doi: 10.1038/s41392-025-02177-z 40320416 PMC12050339

[129] Ma Q Ineichen BV Detmar M Proulx ST . Outflow of cerebrospinal fluid is predominantly through lymphatic vessels and is reduced in aged mice. Nat Commun. (2017) 8:1434. doi: 10.1038/s41467-017-01484-6 29127332 PMC5681558

[130] Wen YR Yang JH Wang X Yao ZB . Induced dural lymphangiogenesis facilities soluble amyloid-beta clearance from brain in a transgenic mouse model of Alzheimer's disease. Neural Regener Res. (2018) 13:709–16. doi: 10.4103/1673-5374.230299 29722325 PMC5950683

[131] Da Mesquita S Papadopoulos Z Dykstra T Brase L Farias FG Wall M . Meningeal lymphatics affect microglia responses and anti-abeta immunotherapy. Nature. (2021) 593:255–60. doi: 10.1038/s41586-021-03489-0 33911285 PMC8817786

[132] Bolte AC Shapiro DA Dutta AB Ma WF Bruch KR Kovacs MA . The meningeal transcriptional response to traumatic brain injury and aging. Elife. (2023) 12:e81154. doi: 10.7554/eLife.81154 36594818 PMC9810333

[133] Mokbel AY Burns MP Main BS . The contribution of the meningeal immune interface to neuroinflammation in traumatic brain injury. J Neuroinflamm. (2024) 21:135. doi: 10.1186/s12974-024-03122-7 38802931 PMC11131220

[134] RebejacJ Eme-ScolanE Arnaud ParoutaudL KharboucheS TelemanM SpinelliL . Meningeal macrophages protect against viral neuroinfection. Immunity. (2022) 55:2103–17:e10. doi: 10.1016/j.immuni.2022.10.005 36323311

[135] Chen J Qin M Xiang X Guo X Nie L Mao L . Lymphocytes in autoimmune encephalitis: pathogenesis and therapeutic target. Neurobiol Dis. (2024) 200:106632. doi: 10.1016/j.nbd.2024.106632 39117118

[136] Miura Y Li MY Birey F Ikeda K Revah O Thete MV . Generation of human striatal organoids and cortico-striatal assembloids from human pluripotent stem cells. Nat Biotechnol. (2020) 38:1421–30. doi: 10.1038/s41587-020-00763-w 33273741 PMC9042317

